# ASH1L in Hepatoma Cells and Hepatic Stellate Cells Promotes Fibrosis‐Associated Hepatocellular Carcinoma by Modulating Tumor‐Associated Macrophages

**DOI:** 10.1002/advs.202404756

**Published:** 2024-10-08

**Authors:** Yuyang Du, Shasha Wu, Shaoyan Xi, Wei Xu, Liangzhan Sun, Jingsong Yan, Han Gao, Yanchen Wang, Jingyi Zheng, Fenfen Wang, Hui Yang, Dan Xie, Xi Chen, Xijun Ou, Xin‐Yuan Guan, Yan Li

**Affiliations:** ^1^ Department of Systems Biology, School of Life Sciences, Southern University of Science and Technology Shenzhen 518055 China; ^2^ Department of Pathology Sun Yat‐Sen University Cancer Center Guangzhou 510275 China; ^3^ GMU‐GIBH Joint School of Life Sciences, The Guangdong‐Hong Kong‐Macau Joint Laboratory for Cell Fate Regulation and Diseases Guangzhou Medical University Guangzhou 511436 China; ^4^ Department of Clinical Oncology The University of Hong Kong Hong Kong 999077 China; ^5^ Institute of Cancer Research Shenzhen Bay Laboratory Shenzhen 518067 China; ^6^ Shenzhen Hospital Southern Medical University Shenzhen 518000 China; ^7^ State Key Laboratory of Oncology in South China and Collaborative Innovation Center for Cancer Medicine Sun Yat‐sen University Cancer Center Guangzhou 510080 China; ^8^ School of Life Sciences Southern University of Science and Technology Shenzhen 518055 China; ^9^ The University of Hong Kong‐Shenzhen Hospital Shenzhen 518053 China

**Keywords:** ASH1L, fibrosis, hepatic stellate cells (HSCs), hepatocellular carcinoma (HCC), macrophage

## Abstract

Hepatocellular carcinoma (HCC) often occurs in the context of fibrosis or cirrhosis. Methylation of histone is an important epigenetic mechanism, but it is unclear whether histone methyltransferases are potent targets for fibrosis‐associated HCC therapy. ASH1L, an H3K4 methyltransferase, is found at higher levels in activated hepatic stellate cells (HSCs) and hepatoma cells. To determine the role of ASH1L in vivo, transgenic mice with conditional *Ash1l* depletion in the hepatocyte cell lineage (*Ash1l^flox/flox^Alb^cre^*) or HSCs (*Ash1l^flox/flox^GFAP^creERT2^*) are generated, and these mice are challenged in a diethylnitrosamine (DEN)/carbon tetrachloride (CCl_4_)‐induced model of liver fibrosis and HCC. Depleting *Ash1l* in both hepatocytes and HSCs mitigates hepatic fibrosis and HCC development. Multicolor flow cytometry, bulk, and single‐cell transcriptomic sequencing reveal that ASH1L creates an immunosuppressive microenvironment. Mechanically, ASH1L‐mediated H3K4me3 modification increases the expression of CCL2 and CSF1, which recruites and polarizes M2‐like pro‐tumorigenic macrophages. The M2‐like macrophages further enhance tumor cell proliferation and suppress CD8^+^ T cell activation. AS‐99, a small molecule inhibitor of ASH1L, demonstrates similar anti‐fibrosis and tumor‐suppressive effects. Of pathophysiological significance, the increased expression levels of mesenchymal ASH1L and M2 marker CD68 are associated with poor prognosis of HCC. The findings reveal ASH1L as a potential small‐molecule therapeutic target against fibrosis‐related HCC.

## Graphical abstract

ASH1L overexpression in activated hepatic stellate and hepatoma cells contributes to the development of fibrosis‐associated hepatocellular carcinoma. ASH1L in stellate cells stimulates fibrogenic gene expression and accelerates fibrosis. Moreover, ASH1L‐mediated H3K4me3 modification increases CCL2 and CSF1 expression in both cell types, which recruits and polarizes macrophages. These tumor‐associated macrophages further enhance tumor cell proliferation and suppress CD8^+^ T cell activation.

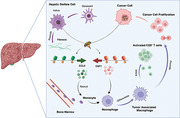

## 1 Introduction

Hepatocellular carcinoma (HCC) is the fifth most prevalent type of cancer and the third leading cause of cancer deaths worldwide.^[^ ^1^ ^]^ Most HCC cases (80–90%) occur in the setting of fibrosis or cirrhosis, irrespective of their different aetiologies.^[^ ^2^, ^3^ ^]^ The incidence of developing HCC is 2–45 times higher in individuals with liver cirrhosis than those in noncirrhotic stages.^[^ ^4^ ^]^ Thus, it is crucial to understand the mechanisms behind fibrosis‐ or cirrhosis‐associated HCC.

Fibrosis and its more advanced form, cirrhosis, involve continuous injury and regeneration of hepatocytes, as well as altered microenvironment with increased stromal stiffness and reduced immune surveillance against tumors.^[^ ^5^, ^6^ ^]^ Among these changes, hepatic stellate cell (HSC) activation is well established as the central driver of cirrhosis‐dependent carcinogenesis. Following liver injury, HSCs become activated, transdifferentiating from vitamin‐A‐storing quiescent cells into proliferative fibrogenic myofibroblasts.^[^ ^7^, ^8^ ^]^ Cell fate tracing also demonstrated that activated HSCs are the major source of cancer‐associated fibroblasts (CAFs) in liver tumors.^[^ ^9^ ^]^ Activated HSCs not only enhance extracellular matrix (ECM) production but also express chemotactic and inflammatory factors to modulate the liver milieu. Genetic depletion or inhibition of activated HSCs in the fibrotic liver strongly suppresses HCC development, indicating that clearing activated HSCs may represent a promising approach to mitigate the risk for HCC development.^[^ ^10^ ^]^

In clinical practice, epigenetic reversion is a more feasible option for clearing activated HSCs than genetic depletion. Histone modifications, DNA methylation, and miRNAs that contribute to HSC activation provide a fertile template for treatment strategies.^[^ ^7^ ^]^ Histone 3 lysine 4 (H3K4) and histone 3 lysine 36 (H3K36) methylations generally impart transcriptional activation effects on fibrogenic genes participating in HSC activation. Several studies have demonstrated the critical roles of histone methyltransferases MLL1, ASH2, and ASH1 in HSC transdifferentiation and liver fibrosis.^[^ ^11^, ^12^, ^13^ ^]^ However, whether and how these enzymes contribute to developing HCC associated with fibrosis or cirrhosis is currently unclear. In the present study, we set out to identify histone methyltransferases that are expressed at higher levels in activated HSCs and hepatoma cells, compared to quiescent stellate cells and normal hepatocytes. It was found that ASH1 Like Histone Lysine Methyltransferase (ASH1L) meets this criterion, suggesting its potential involvement in HSC activation and the related development of HCC. In vitro coculture systems and in vivo Cre‐LoxP conditional knockout mouse models were used to investigate the roles of HSC‐specific and hepatocyte‐specific ASH1L activities in fibrosis‐associated HCC.

## 2 Results

### 2.1 Simultaneous Depletion of *Ash1l* in Hepatocytes and HSCs Mitigate HCC and Fibrosis Development In Vivo

**Figure 1:**
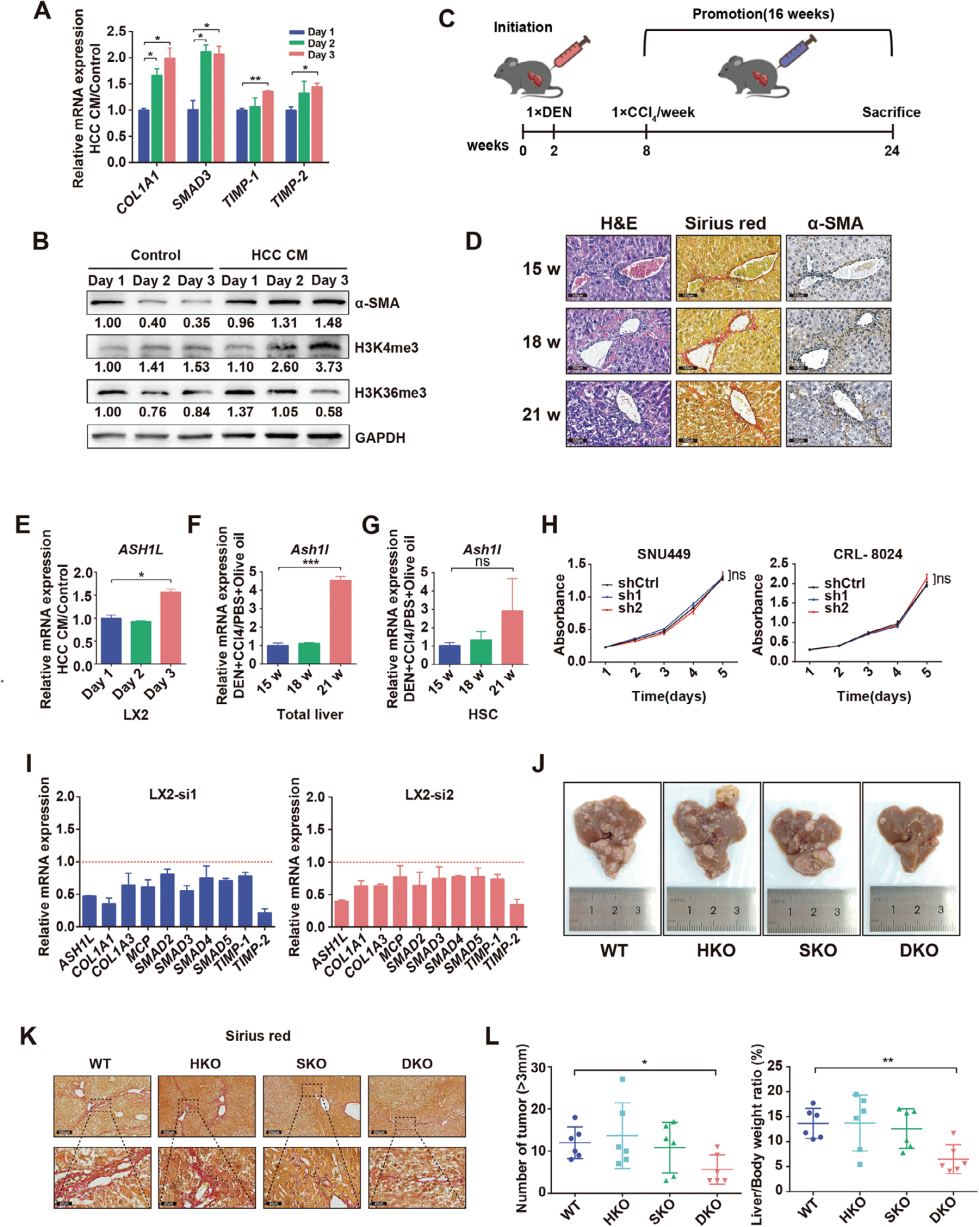
ASH1L expression is positively correlated with HSC activation and HCC development. A) RT‐qPCR measured HSC activation marker genes expression post‐HCC‐CM treatment. B) α‐SMA, H3K4me3, and H3K36me3 levels in LX2 HSCs post‐HCC‐CM treatment were analyzed using Western blots. C,D) Schematic summary (C) of the DEN/CCl_4_‐induced mouse model of HCC. Livers staining with H&E, Sirius red, and anti‐α‐SMA (D) at week 15, 18, and 21. Scale bars, 100 µm. E) RT‐qPCR assessed *ASH1L* expression in LX2 post‐HCC‐CM treatment. F,G) RT‐qPCR assessed *Ash1l* expression in total liver (F) and isolated HSCs (G) of the DEN/CCl_4_ mice versus control. H) Viability of human HCC cell lines was assessed using CCK‐8 assay. I) Expression of HSC activation marker genes in LX2 cells after ASH1L silencing with two independent siRNA sequences was detected using RT‐qPCR. J–L)WT, HKO, SKO, and DKO mice were sacrificed at week 24 upon DEN/CCl_4_‐induced hepatocarcinogenesis. Gross liver images (J), Sirius red staining of livers (K), tumor numbers and liver‐to‐body weight ratio (L) of the indicated groups were presented (*n* = 6). Scale bars, 200 µm (upper panel); 40 µm (lower panel). Data are presented as mean ± SD. *P* values were computed using the unpaired Student's *t*‐test. ^∗^ *P*  < 0.05, ^∗∗^ *P* < 0.01, ^∗∗∗^ *P *< 0.001. ns. not significant.

To imitate the activation of HSCs in the tumor microenvironment, the human HSC cell line LX2 was cultured with the conditioned medium (CM) of human HCC cell line CRL‐8024. This treatment increased the expression of HSC activation marker genes, as well as the levels of trimethylated H3K4 (H3K4me3) and most H3K4me3 methyltransferases, including *ASH1L* (**Figure** **1**A,B,E; Figure S1A, Supporting Information). In vivo, a fibrosis‐associated HCC mouse model was induced and the Sirius red (SR) and α‐SMA staining results verified that collagen fiber deposition and HSC activation increase progressively with the development of HCC (Figure 1C,D). Among the various H3K4me3 methyltransferases, the expression of *Ash1l*, *Kmt2a*, and *Smyd3* was elevated in the liver at week 21 when fibrosis and HCC formed (Figure 1F; Figure S1B, Supporting Information). Mouse hepatocytes and HSCs were further isolated and the RT‐qPCR results showed that *Ash1l* was highly expressed in the HSCs of the advanced stage of hepatocarcinogenesis (Figure 1G; Figure S1C,D, Supporting Information). Collectively, ASH1L is an H3K4me3 methyltransferase that consistently shows increased expression in activated HSCs and tumor cells both in vitro and in vivo.

To investigate the impact of ASH1L on the development of HCC and its associated HSC activation, we created HCC and HSC cell lines with ASH1L knockdown (Figure S1E,F, Supporting Information). The CCK‐8 assay revealed that the knockdown of ASH1L in tumor cells did not significantly affect cell proliferation (Figure 1H). However, in LX2 HSCs, the knockdown of ASH1L resulted in decreased expression of HSC activation marker genes and profibrogenic genes, indicating that ASH1L promotes HSC activation (Figure 1I). In vivo, we crossed *Ash1l ^flox/flox^* mice with *Albumin‐Cre* (*Alb‐Cre)* or *Gfap‐CreERT2* to generate three different types of knockout mice: hepatocyte‐specific *Ash1l* knockout mice (HKO), HSC‐specific *Ash1l* knockout mice (SKO), and double knockout mice (DKO). The *Ash1l ^flox/flox^* mice were used as wild‐type (WT) controls (Figure S1G,H, Supporting Information). We then introduced the DEN/CCl_4_‐induced HCC model in all four groups of mice to study the impact of *Ash1l* on the development of fibrosis‐associated HCC (Figure 1J). Sirius red staining showed that both HSC‐specific deletion and double deletion of *Ash1l* could effectively inhibit fibrosis, consistent with the in vitro results of LX2 cells (Figure 1K). Regarding tumor development, the DKO group had lower tumor numbers and liver‐to‐body weight ratios than the WT group, while there were no significant differences between HKO, SKO, and WT groups (Figure 1L). These results suggest that HSC‐specific *Ash1l* deletion can ameliorate hepatic fibrosis, while simultaneous depletion of *Ash1l* in both hepatocytes and HSCs is necessary to mitigate HCC development in vivo.

### 2.2 ASH1L Creates an Immunosuppressive Micro‐Environment in Fibrosis‐Associated HCC

**Figure 2:**
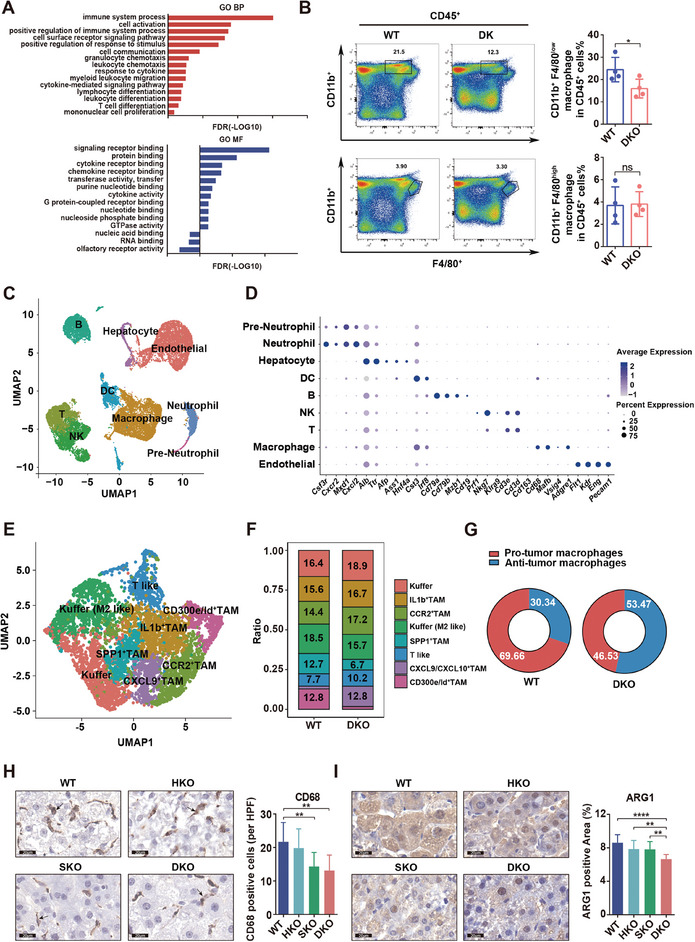
ASH1L expression in hepatocytes and HSCs creates an immune‐suppressive micro‐environment in fibrosis‐associated HCC. A) Gene Ontology (GO) functional enrichment analysis of differentially expressed genes between WT and DKO mouse tumor tissues. B) The proportion of macrophages in the liver tumor tissues of WT and DKO mice was detected using multicolor flow cytometry (*n* = 4). C) UMAP projection of single cells derived from WT and DKO mouse liver tissues colored by cell clusters. D) Bubble heatmap depicting the expression levels of cluster‐specific marker genes. E) UMAP representation of macrophage subclusters. F) Scale diagram showing different macrophage subcluster compositions in WT and DKO mice. G) Percentage of pro‐tumor and anti‐tumor macrophages in WT and DKO mice. H–I) IHC staining of M2 marker (CD68, ARG1) in mouse HCC tissues. Scale bars, 20 µm. Three photographs were obtained from each mouse (*n* = 3). Data are presented as mean ± SD. *P* values were computed using the unpaired Student's *t*‐test (H, I) and paired Student's *t*‐test (B). ^∗^ *P* < 0.05, ^∗∗^ *P* < 0.01, ^∗∗∗∗^ *P* < 0.0001. ns. not significant.

Transcriptome sequencing was conducted on transgenic mouse tissues to uncover the mechanisms behind ASH1L‐mediated fibrosis and HCC development. The Gene Ontology (GO) functional enrichment analysis of the biological process (BP) and molecular function (MF) showed that the differentially expressed genes between DKO and WT groups were mainly enriched in the immune system process mediated by cytokines and their receptors (**Figure** **2**A). To evaluate immune cell composition in DKO and WT mice, multicolor flow cytometry was then applied. The results revealed that the proportion of recruited bone marrow (BM)‐derived macrophages (CD11b^+^F4/80^low^), but not yolk sac‐derived residential macrophage/Kupffer cells (CD11b^+^F4/80^high^), was reduced in DKO mice compared with WT mice (Figure 2B). The proportion of other immune cells did not show significant changes (Figure S2A, Supporting Information). Hence, the flow cytometry results suggested that ASH1L might affect the recruitment of BM‐derived macrophages. Single‐cell RNA sequencing (scRNA‐seq) was further used to comprehensively investigate the transcriptomic profiles of individual cell populations in transgenic mouse tumor tissues. UMAP dimensionality reduction analysis identified 9 clusters, corresponding to established cell‐type markers in mouse liver, including endothelial cells, macrophages, T cells, natural killer (NK) cells, B cells, dendritic cells (DCs), neutrophil cells, pre‐neutrophil cells, and hepatocytes (Figure 2C,D). The macrophage cluster is the largest immune cell population, accounting for approximately 24% of the total cell fraction (Figure S2B, Supporting Information). The subcluster analysis of macrophages revealed eight subpopulations, which were subsequently assigned names based on their distinct transcriptional signatures (Figure 2E; Figure S2C, Supporting Information). Growing evidence suggests that tumor‐associated macrophages (TAMs) are key components of the complex tumor microenvironment (TME), which can exhibit either pro‐tumorigenic or anti‐tumorigenic functions depending on their diverse subpopulations and intricate heterogeneity.^[^ ^14^, ^15^, ^16^, ^17^ ^]^ ScRNA‐seq results showed that the proportion of pro‐tumorigenic TAM subpopulations, represented by SPP1^+^ TAM and CD300e/Id^+^ TAM, decreased, while the proportion of anti‐tumorigenic TAM cells, represented by CXCL9^+^ TAM, increased in the DKO group as compared to WT group (Figure 2F,G). Immunohistochemistry (IHC) staining confirmed that M2 macrophage markers CD68 and ARG1 were lower in the DKO mice than in WT mice (Figure 2H,I). Additionally, T cell subcluster analysis showed that there was a higher proportion of T helper cells and activated CD8^+^ T cells (CD8ac) in the DKO group than in the WT group (Figure S2D–F, Supporting Information). Collectively, these observations suggest that ASH1L expression in hepatocytes and HSCs creates an immunosuppressive microenvironment by affecting M2‐like pro‐tumorigenic TAMs and active CD8^+^ T cells composition.

### 2.3 Expression of ASH1L in Hepatocytes and HSCs Regulates Macrophage Recruitment and M2 Polarization

**Figure 3:**
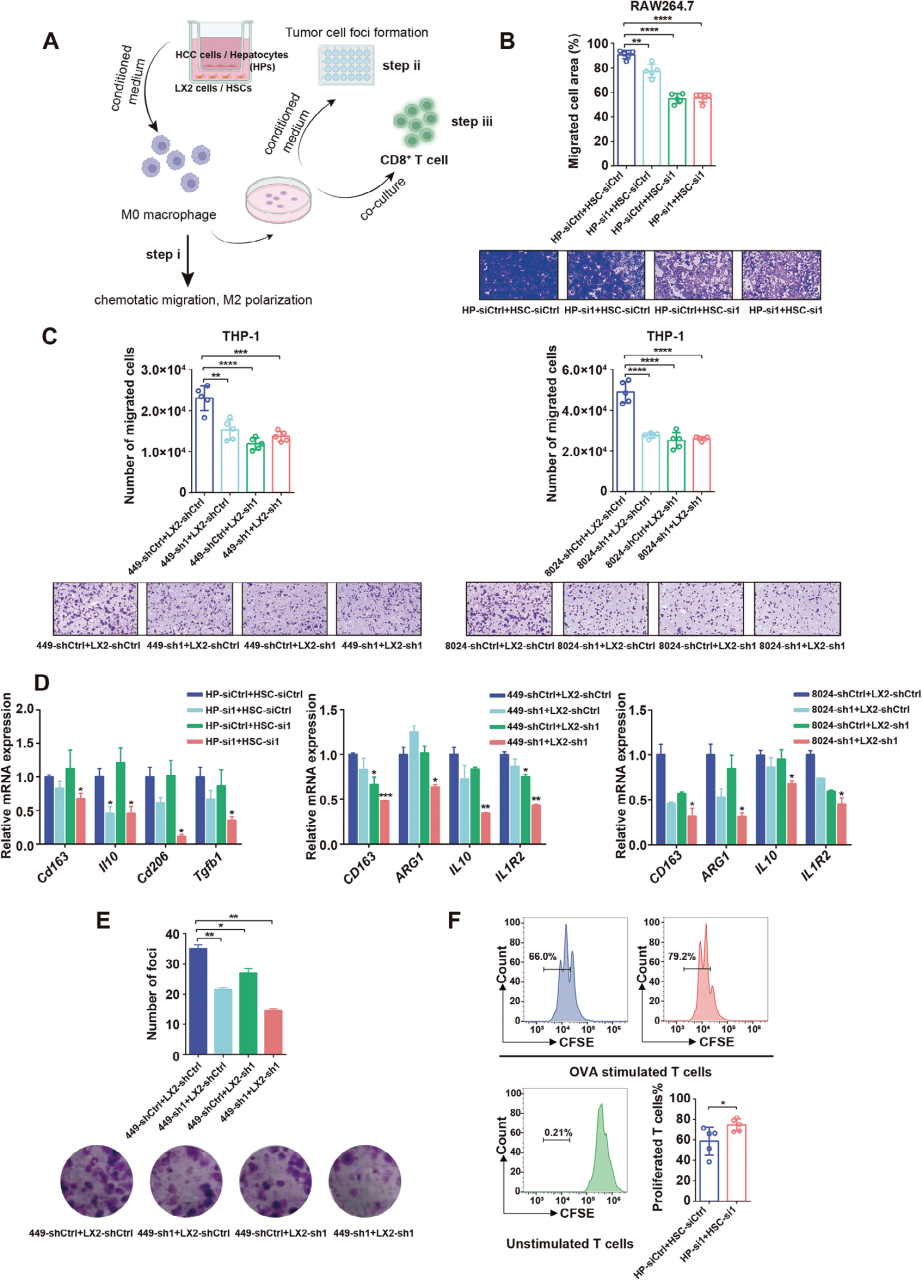
Expression of ASH1L in hepatocytes and HSCs regulates macrophage recruitment and M2 polarization. A) Schematic diagram of coculture experiments. Step i: B–D, Step ii: E, Step iii: F, HPs: Hepatocytes. B) Chemotactic migration assays of Raw264.7 mouse macrophage cell line using CM from the indicated mouse primary hepatocytes and HSCs (*n* = 5). C) Chemotactic migration assays of human monocyte line THP‐1 using CM of cocultured human HCC cell lines SNU449 /CRL‐8024 and HSC cell line LX2 as indicated (*n* = 5). D) RT‐qPCR for M2 macrophage markers expression after treatment with the indicated CM. E) Representative images and quantification of tumor cell foci when cultured with CM from macrophages polarized by cocultured SNU449 and LX2 cells as indicated (*n* = 3). F) CFSE histograms detected the proliferation of CD8^+^ T cell cocultured with macrophages that induced by CM of the indicated primary hepatocytes and HSCs (*n* = 5). Data are presented as mean ± SD. *P* values were computed using the unpaired Student's *t*‐test. ^∗^ *P* < 0.05, ^∗∗^ *P* < 0.01, ^∗∗∗^ *P *< 0.001, ^∗∗∗∗^ *P* < 0.0001.

Primary cultures of mouse hepatocytes, HSCs, and human HCC and HSC cell lines were used to investigate the effect of hepatocyte‐ and HSC‐specific ASH1L on macrophage recruitment and polarization. Hepatocytes and HSCs were cocultured in transwell chambers, and the conditioned medium was then collected and used to treat M0 macrophages (**Figure** **3**A). Knocking down ASH1L in either HSCs or tumor cells could inhibit macrophage recruitment and reduce M2 macrophage marker genes expression, with the strongest effect seen in the double knockdown group (Figure 3B–D; Figure S3A–D, Supporting Information). We confirmed our observations in both murine primary cells and human cell line systems. As M2‐like pro‐tumorigenic TAMs are known to promote cancer cell proliferation and inhibit CD8^+^ T cell activation, we continued to examine whether ASH1L‐polarized macrophages change their functional properties. The CM of polarized macrophages was collected to culture SNU449 HCC cells. Foci formation assay demonstrated that double knockdown of ASH1L in tumor cells and HSCs induced polarized macrophages with the highest ability to inhibit tumor growth, as compared to polarized macrophages induced by single knockdown of ASH1L in either HCC or HSC (Figure 3E; Figure S3E, Supporting Information). We also evaluated CM from ASH1L‐polarized macrophages on T cell proliferation and IFN‐γ production. Labeling CD8^+^ T cells with carboxyfluorescein succinimidyl ester (CFSE) fluorescent dye, we found that double knockdown of ASH1L in tumor cells and HSCs induced polarized macrophages that promoted CD8^+^ T cell proliferation (Figure 3F; Figure S3F, Supporting Information). Moreover, no difference was observed between control and double knockdown groups in influencing ovalbumin (OVA)‐specific CD8^+^ cytotoxic OT‐1 T cells IFN‐γ production (not shown here). Taken together, these results suggest that ASH1L expression in hepatocytes and HSCs promotes tumor cell proliferation and inhibits CD8^+^ T cell activation via macrophage recruitment and M2 polarization.

### 2.4 ASH1L Regulates the Expression of CCL2 and CSF1 in Both Hepatocytes and HSCs

**Figure 4:**
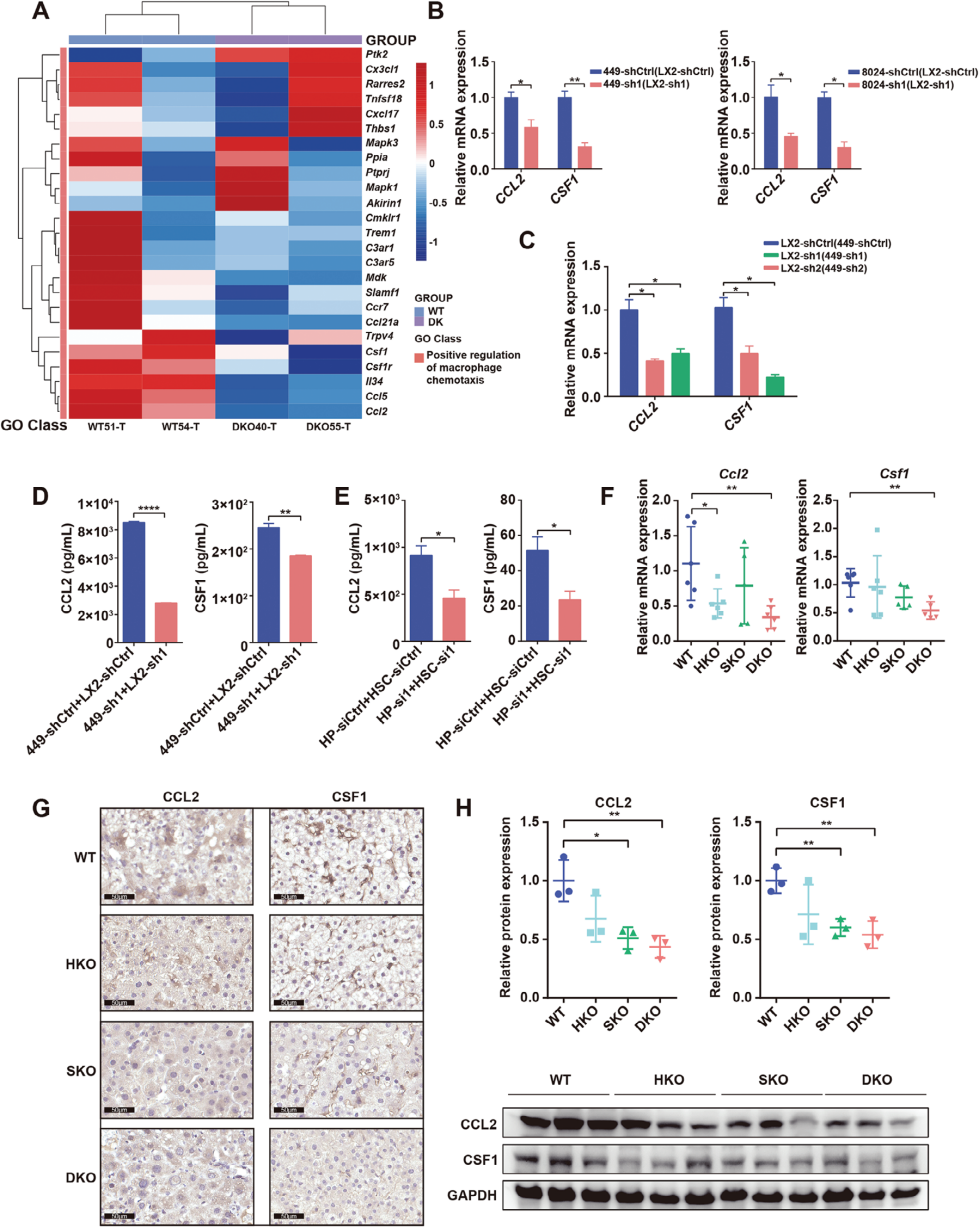
ASH1L regulates the expression of CCL2 and CSF1 in both hepatocytes and HSCs. A) Heatmap displays the differentially expressed genes between WT and DKO transgenic mouse liver tumor tissues. These genes positively regulate macrophage chemotaxis and the color bar represents relative gene expression magnitude (*Z*‐score normalization) (*n* = 2). B) *CCL2* and *CSF1* mRNA expression in ASH1L knockdown HCC cell lines SNU449 (left) and CRL‐8024 (right). (LX2‐shCtrl): cocultured with control LX2 cells, (LX2‐sh1): cocultured with sh1‐mediated ASH1L knockdown LX2 cells. C) *CCL2* and *CSF1* mRNA expression in ASH1L knockdown LX2 cells. (449‐shCtrl): cocultured with control SNU449 cells, (449‐sh1)/(449‐sh2): cocultured with sh1/sh2‐mediated ASH1L knockdown SNU449 cells. D–E) ELISA assay detected secretory CCL2 and CSF1 in conditioned medium of cocultured SNU449 + LX2 cells (D) or primary mouse hepatocytes (HP) + HSCs (E) as indicated. (F) *Ccl2* and *Csf1* mRNA expression in the liver tissues of the indicated transgenic mice (*n* = 6). G) IHC staining of CCL2 and CSF1 in the liver tumor tissues of the indicated transgenic mice. Scale bars, 50 µm. H) Western blot analysis assessed CCL2 and CSF1 protein expression in the liver tissues of the indicated transgenic mice (*n* = 3). Data are presented as mean ± SD. *P* values were computed using the unpaired Student's *t*‐test. ^∗^ *P* < 0.05, ^∗∗^ *P* < 0.01, ^∗∗∗∗^ *P* < 0.0001.

We then continued to identify downstream effectors that mediate ASH1L's regulation of macrophage recruitment and polarization. Candidates were screened from differentially expressed genes between DKO mice and WT mice that enriched in positive regulation of macrophage chemotaxis function (**Figure** **4**A). *CCL2* and *CSF1* were validated as the most significantly downregulated genes in the human HCC and HSC coculture system. Upon ASH1L double knockdown, *CCL2* and *CSF1* expression decreased in cocultured SNU449/CRL‐8024 HCC cells and LX2 HSCs (Figure 4B,C; Figure S4A,B, Supporting Information). We confirmed these observations using siRNA to silence *Ash1l* in murine primary hepatocytes and HSCs (Figure S4C,D, Supporting Information). As CCL2 and CSF1 are secretory factors, the enzyme‐linked immunosorbent assay (ELISA) was utilized to assess their protein levels in both human cell lines and murine primary cells coculture systems. Consistent with their mRNA levels, the secretory CCL2 and CSF1 were reduced in CM of ASH1L double knockdown group (Figure 4D,E). In vivo, the correlation between ASH1L and CCL2 as well as CSF1 was examined with RT‐qPCR, IHC and western blot of transgenic mice tissues. The expression of CCL2 and CSF1 declined after ASH1L knockdown in either HKO or SKO mice, the effect of which became more evident in the double knockdown group (Figure 4F–H). These results indicate that ASH1L regulates the expression of CCL2 and CSF1 in both hepatocytes and HSCs.

### 2.5 ASH1L‐Mediated H3K4me3 Modification Increases CCL2 and CSF1 Expression to Recruit and M2‐Polarize Macrophages

**Figure 5:**
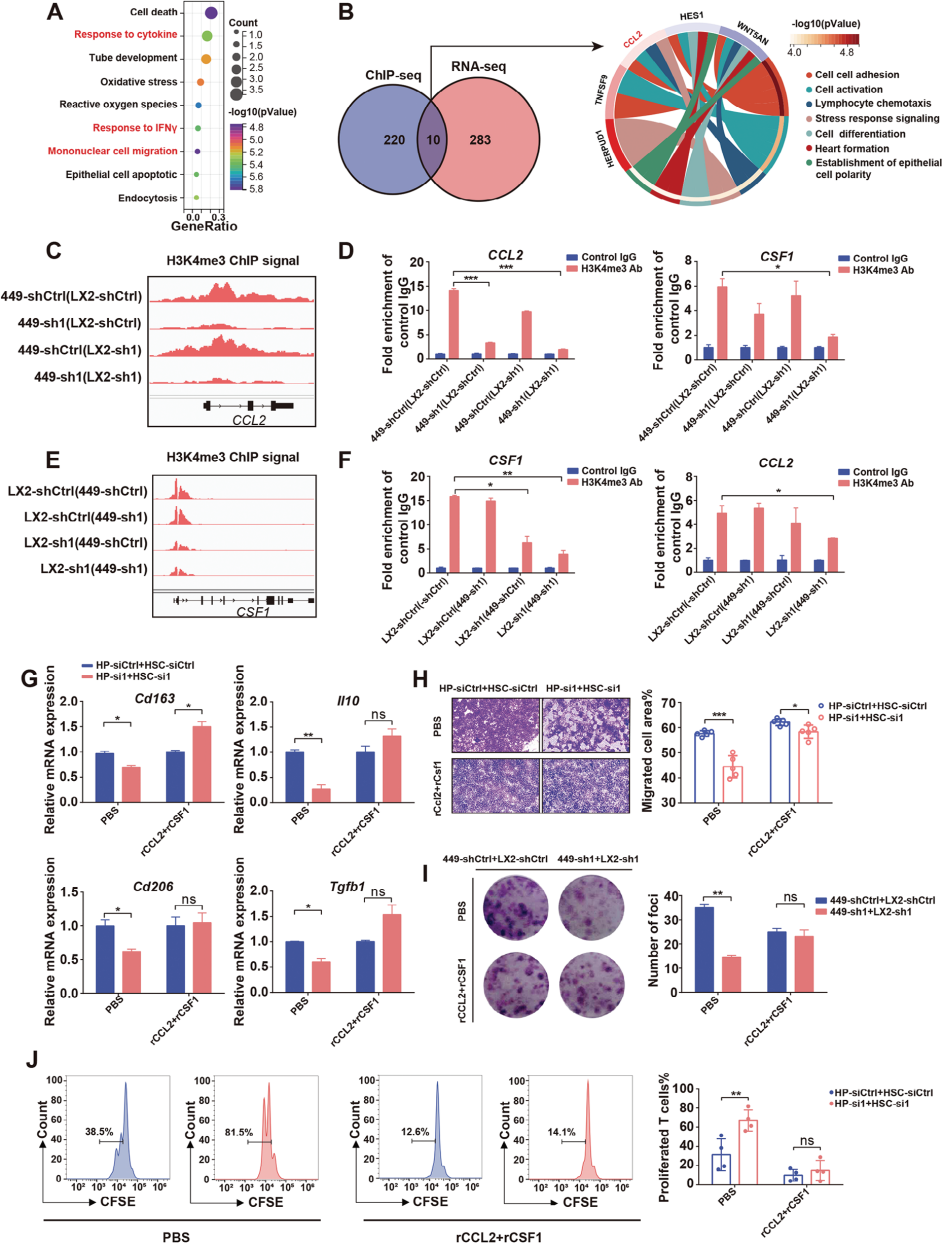
ASH1L‐mediated H3K4me3 modification increases CCL2 and CSF1 expression to recruit and M2‐polarize macrophages. A) Gene Ontology (GO) biological process enrichment analysis of the genes with down‐regulated H3K4me3 peaks at promoters in ASH1L knockdown SNU449 cells (cocultured with ASH1L knockdown LX2 cells). B) Venn plots (left) of genes with down‐regulated H3K4me3 peaks at promoters ChIP‐seq data and RNA‐seq data in SNU449‐sh1(LX2‐sh1) versus SNU449‐shCtrl(LX2‐shCtrl). Chord diagram (right) showing the core genes of signaling pathways. C) ChIP‐seq profile of differential H3K4me3 peaks in the *CCL2* gene of SNU449 cells cocultured with LX2 cells as indicated. D) ChIP‐qPCR analysis of IgG, and anti‐H3K4me3 antibody at *CCL2* and *CSF1* promoters in SNU449 cells cocultured with LX2 cells as indicated. E) ChIP‐seq profile of differential H3K4me3 peaks in the *CSF1* gene of LX2 cells cocultured with SNU449 cells as indicated. F) ChIP‐qPCR analysis of IgG, and anti‐H3K4me3 antibody at *CSF1* and *CCL2* promoters in LX2 cells cocultured with SNU449 cells as indicated. G) RT‐qPCR detected M2 markers expression in macrophages treated with the CM of cocultured primary hepatocytes and HSCs. The conditioned medium was supplemented with either PBS or 50 ng mL^−1^ of mouse recombinant proteins rCCL2 and rCSF1 (rCCL2 + rCSF1). H) Chemotactic migration assays of macrophages using the indicated CM of cocultured primary hepatocytes and HSCs (*n* = 5). I) Representative images and quantification of tumor cell foci when cultured with the indicated CM from polarized macrophages induced by the cocultured SNU449 and LX2 cells (*n* = 3). J) CFSE histograms detected the proliferation of CD8^+^ T cells cocultured with macrophages induced by the indicated CM of primary hepatocytes and HSCs (*n* = 4). Data are presented as mean ± SD. *P* values were computed using the unpaired Student's *t*‐test (D, F, G, H, I) and paired Student's *t*‐test (J). ^∗^ *P* < 0.05, ^∗^ *P* < 0.01, ^∗∗∗^ *P *< 0.001. ns. not significant.

It is known that ASH1L enhances target gene expression by directly binding to the gene promoters and modifying H3K4me3 levels.^[^ ^18^, ^19^, ^20^, ^21^ ^]^ Chromatin immunoprecipitation (ChIP)‐sequencing was performed to profile genome‐wide patterns of H3K4me3 in cocultured SNU449 HCC cells and LX2 HSCs. We detected significantly reduced H3K4me3 levels in 220 gene promoters of cocultured SNU449 HCC cells of the DKO group compared to that in the control group. GO term enrichment analysis of the genes with down‐regulated H3K4me3 peaks in promoters, revealed statistically significantly enriched cytokine response, IFNγ response, and mononuclear cell migration pathways that related to immune regulation (**Figure** **5**A). Integrating this H3K4me3 ChIP‐seq data with RNA‐seq data, we found 10 overlapped genes that were also downregulated at transcriptional level, among which *CCL2* plays an essential role in immune cell recruitment and activation (Figure 5B). Visualization of ChIP‐seq data showed a decrease in H3K4me3 occupancy of *CCL2* and *CSF1* promoter in SNU449 cells upon ASH1L knockdown (Figure 5C). These effects on H3K4me3 methylation were further validated by analysis using ChIP with quantitative PCR (ChIP–qPCR) (Figure 5D). Cocultured LX2 HSCs also exhibited concordant changes of H3K4me3 modification at *CSF1* and *CCL2* promoter regions after silencing ASH1L (Figure 5E,F). In particular, H3K4me3 signal reduction in tumor cells was further exacerbated when ASH1L was knocked down in the cocultured HSCs and vice versa. This implies that tumor cells and HSCs may crosstalk with each other to strengthen the synergetic effect.

To determine whether the effects of ASH1L on macrophage recruitment and polarization are dependent on CCL2 and CSF1, mouse recombinant CCL2 and CSF1 were added to coculture CM of murine primary hepatocytes and HSCs where ASH1L had been silenced. Replenishing the recombinant CCL2 and CSF1 (rCCL2+rCSF1) reversed the suppression of macrophage M2 polarization and recruitment caused by ASH1L ablation (Figure 5G,H; Figure S5A,S6A, Supporting Information). Similar results were obtained in coculture systems of human HCC cells and HSCs (Figure S5B,S6B,C, Supporting Information). The addition of recombinant CCL2 and CSF1 (rCCL2+rCSF1) supplements also reversed the HCC cell proliferation inhibition and CD8^+^ T cell activation phenotypes caused by ASH1L knockdown (Figure 5I,J; Figure S7A,B, Supporting Information). These findings suggest that ASH1L‐mediated H3K4me3 modification increases the expression of CCL2 and CSF1, leading to the recruitment and M2‐polarization of macrophages.

### 2.6 The Small Molecule AS‐99 Demonstrates Similar Tumor Suppressive Effects as the Genetic Depletion of ASH1L

**Figure 6:**
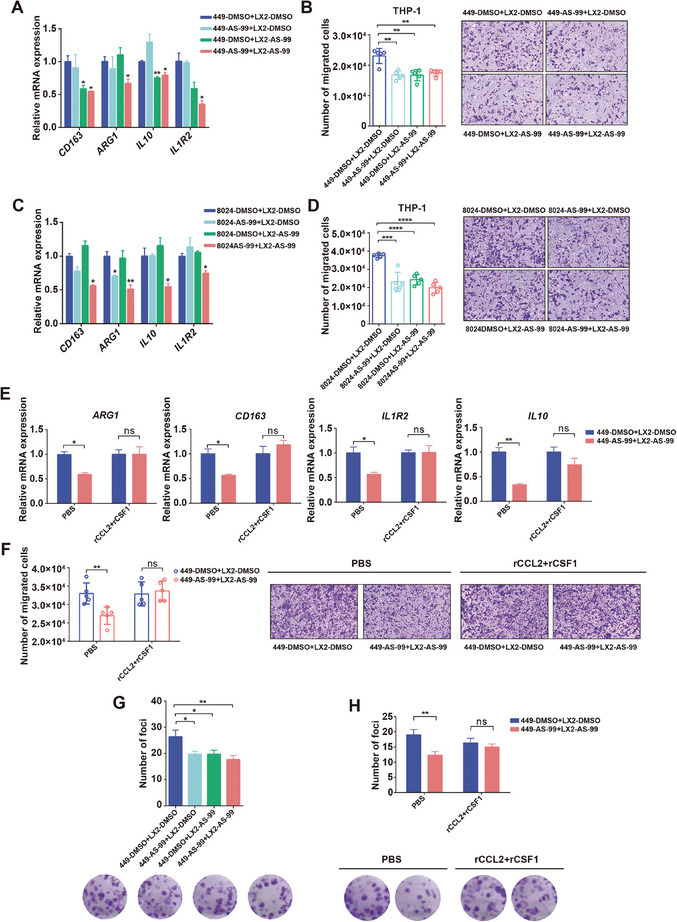
The small molecule AS‐99 demonstrates similar tumor suppressive effects as the genetic depletion of ASH1L. A,C) RT‐qPCR detected M2 markers expression in macrophages treated with the CM from cocultured SNU449 and LX2 (A) or cocultured CRL‐8024 and LX2 (C). SNU449, CRL‐8024 and LX2 cells were treated with AS‐99 or DMSO for 7 days prior to coculture. B,D) Chemotactic migration assays of macrophages using the CM from cocultured SNU449 and LX2 (B) or cocultured CRL‐8024 and LX2 (D) (*n* = 5). E) RT‐qPCR detected expression of M2 markers in macrophages treated with the CM from cocultured SNU449 and LX2. The conditioned medium was supplemented with either PBS or human recombinant proteins rCCL2 and rCSF1 (rCCL2 + rCSF1). F) Chemotactic migration assays of macrophages using the indicated CM of cocultured SNU449 and LX2 (*n* = 5). G, H) Representative images and quantification of tumor cell foci when cultured with the indicated CM from polarized macrophages induced by the cocultured SNU449 and LX2 cells (*n* = 3). Data are presented as mean ± SD. *P* values were computed using the unpaired Student's *t*‐test. ^∗^ *P* < 0.05, ^∗∗^ *P* < 0.01. ns. not significant.

To investigate the potential of targeting ASH1L as a clinical intervention, we evaluated AS‐99, a recently developed small molecule inhibitor of the ASH1L, on tumor cells and HSCs.^[^ ^22^ ^]^ Upon treatment with AS‐99, the expression of HSC activation marker genes and profibrogenic genes decreased in LX2 HSCs (Figure S8A, Supporting Information), which was consistent with the results of ASH1L knockdown. The AS‐99 also reduced the H3K4me3 modification and expression of *CSF1* and *CCL2* in both tumor cells and HSCs (Figure S8B,C, Supporting Information). To test whether AS‐99 affects macrophage recruitment and polarization, SNU449 HCC and LX2 cells were treated with AS‐99 or dimethyl sulfoxide (DMSO) for 7 days, followed by coculturing in transwell chambers. The coculture CM was then collected to induce M0 macrophage recruitment and polarization. The expression of the M2 polarization markers and macrophage recruitment were inhibited after AS‐99 treatment in either tumor cells or HSCs, with stronger effects when both cells were treated with AS‐99 (**Figure** **6**A,B). The pro‐proliferative ability of polarized macrophages was abated after AS‐99 treatment (Figure 6G). We observed similar results when applying AS‐99 in another HCC cell line CRL‐8024 that cocultured with LX2 (Figure 6C,D). We continued to test whether inhibiting CCL2 and CSF1 is essential for AS‐99 activity, just as it does for ASH1L knockdown. Adding recombinant CCL2 and CSF1 proteins reversed the inhibition of macrophage M2 polarization, recruitment and tumor proliferation phenotypes caused by AS‐99 treatment (Figure 6E,F,H; Figure S8D,E, Supporting Information). These results suggest that AS‐99 exerts similar tumor suppressive effects as genetic depletion of ASH1L through inhibiting macrophage recruitment and M2 polarization.

### 2.7 Therapeutic Potential of Inhibiting ASH1L and Its Downstream Effectors in HCC

**Figure 7:**
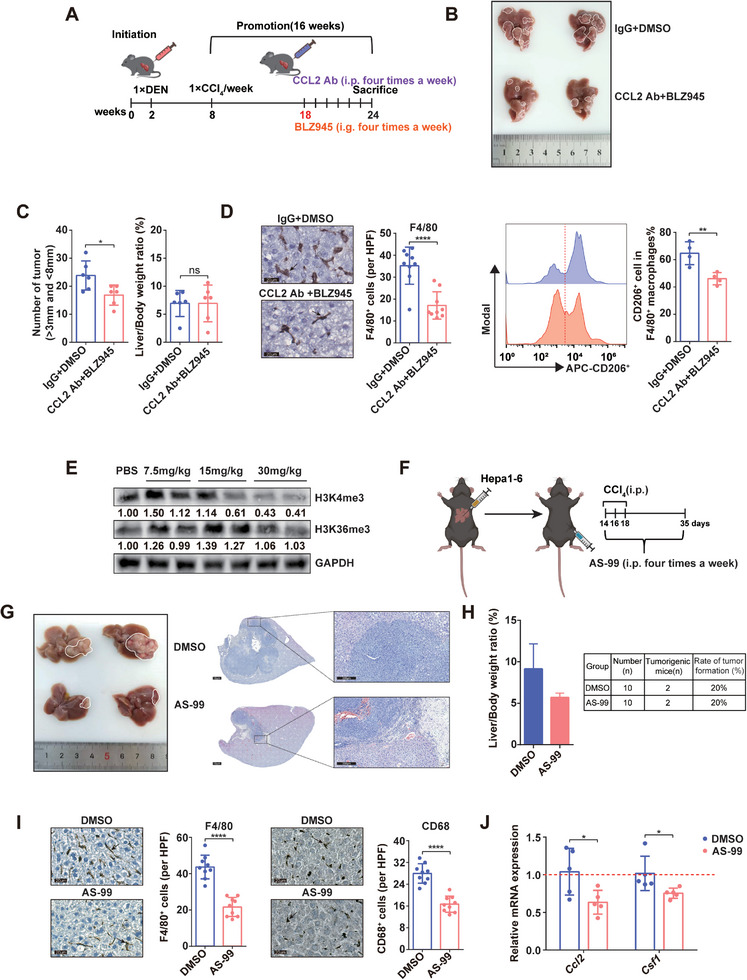
Therapeutic potential of ASH1L inhibition and downstream pathway blockade in HCC. A) Schematic diagram of the treatment of DEN/CCl_4_‐induced HCC mouse model. B–D) Mice were sacrificed at week 24 upon DEN/CCl_4_‐induced hepatocarcinogenesis. Gross liver images (B), tumor numbers and liver‐to‐body weight ratio (*n* = 6) (C), IHC staining of F4/80. Three photographs were obtained from each mouse (n = 3) (D), flow cytometry of M2 marker CD206 (*n* = 4) of the indicated groups were presented. E) The changes of methylation of histone in liver of mice under different AS‐99 doses were detected by western blots. F) Schematic diagram of AS‐99 administration in mouse orthotopic liver cancer model. G) Gross liver images (left) and HE staining (right) results. H) Statistical analysis of liver‐to‐body weight ratio and orthotopic tumor formation rate in mice. I) IHC staining results and statistical analysis of F4/80 (left) and M2 Marker CD68(right) in mouse liver tissue. Three photographs were obtained from each mouse (n = 3). Scale bars, 20 µm. (J) *Ccl2* and *Csf1* mRNA expression in mouse liver tissue (*n* = 5). Data are presented as mean ± SD. *P* values were computed using the unpaired Student's *t*‐test. ^∗^ *P* < 0.05, ^∗∗^ *P* < 0.01, ^∗∗∗∗^ *P* < 0.0001.

To evaluate the therapeutic potential of ASH1L inhibition in HCC, we utilized a CCL2‐neutralizing antibody (CCL2 Ab) and the CSF1R inhibitor BLZ945 to block CCL2 and CSF1 signaling in a DEN/CCl_4_‐induced mouse model. At 18 weeks post‐DEN‐CCl_4_ administration, fibrotic lesions began to form in the mouse liver, indicating HSC activation and upregulation of ASH1L expression (Figure 1D). The combined treatment with CCL2 Ab and BLZ945 was then administered (**Figure** **7**A). Compared to the IgG+DMSO control group, this combination therapy significantly reduced tumor numbers without affecting liver‐to‐body weight ratios (Figure 7B–C). IHC and flow cytometry analyses revealed that CCL2 Ab and BLZ945 treatment inhibited F4/80^+^ macrophage infiltration and reduced M2 macrophage marker CD206 expression (Figure 7D). These findings suggest that targeting the CCL2 and CSF1 signaling pathways downstream of ASH1L is a feasible strategy to suppress liver cancer progression in a fibrotic microenvironment.

Additionally, we investigated the in vivo therapeutic effects of the ASH1L inhibitor AS‐99. To determine the optimal dosage, C57BL/6 mice received three consecutive injections of AS‐99 at varying doses. Western blot analysis indicated that a 30 mg kg^−1^ dose significantly inhibited H3K4me3 modification in liver tissue, with minimal impact on H3K36me3 modification (Figure 7E). An orthotopic liver cancer model was established by injecting Hepa1‐6 cells into the livers of C57BL/6 mice. Fourteen days post‐injection, fibrosis was induced via three consecutive CCl_4_ injections. AS‐99 was administered intraperitoneally until sacrifice at 35 days post‐tumor inoculation (Figure 7F). AS‐99 treatment markedly inhibited in situ HCC growth (Figure 7G). Although a downward trend in liver‐to‐body weight ratio was observed in the AS‐99‐treated group, the low tumor formation rate in this model precluded sufficient comparison (Figure 7H). IHC staining of liver tissues from nontumorigenic mice demonstrated significant inhibition of F4/80^+^ and CD68^+^ macrophage infiltration in all treated mice after AS‐99 treatment (Figure 7I). These findings suggest that AS‐99 not only suppresses HCC growth but also impedes the recruitment and polarization of macrophages within the liver microenvironment, even in the absence of liver tumors. RT‐qPCR analysis further confirmed that AS‐99 treatment suppressed CCL2 and CSF1 expression, corroborating the regulatory role of ASH1L in these chemokines (Figure 7J). Collectively, these results suggest that blocking the CCL2‐CSF1 signaling pathway downstream of ASH1L or directly inhibiting ASH1L enzymatic activity holds promise as a clinical approach for treating ASH1L‐mediated HCC.

### 2.8 ASH1L Expression Linked to Elevated M2 Marker CD68 Levels and Poor Prognosis in HCC

**Figure 8:**
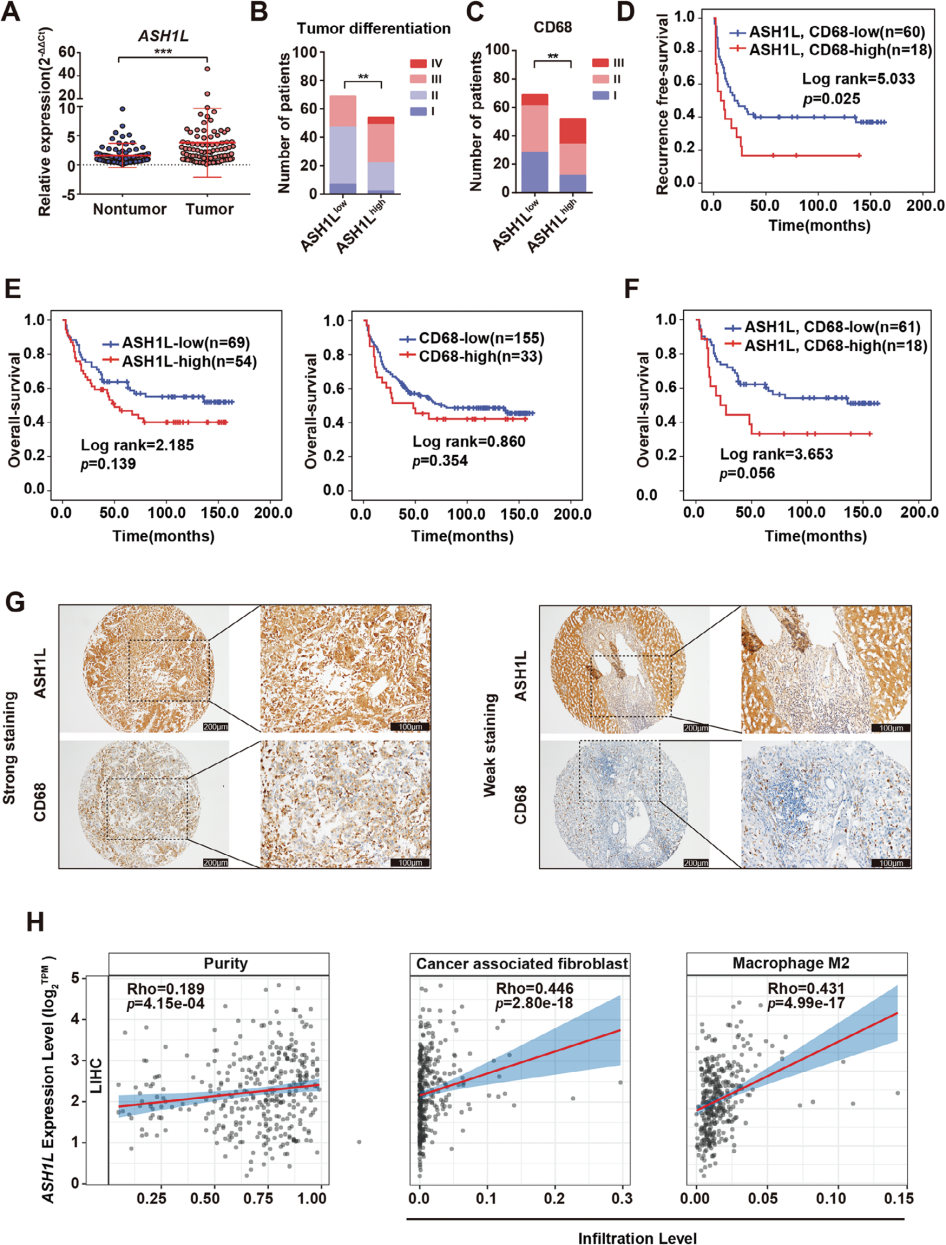
Clinical relevance of ASH1L and HCC progression. A) RT‐qPCR analyzed *ASH1L* mRNA expression in our in‐house HCC tumor tissues and paired nontumor tissues (cohort 1) (n = 99). B,C) ASH1L protein levels in mesenchyma were correlated with histological differentiation (*n* = 123) (B) and M2 marker levels (*n* = 121) (C). D) Kaplan–Meier recurrence‐free survival curves of HCC patients with normal versus high mesenchymal ASH1L and CD68. E) Kaplan–Meier overall survival curves of HCC patients with normal versus high ASH1L (left) or high CD68 (right). F) Kaplan–Meier overall survival curves of HCC patients with normal versus high mesenchymal ASH1L and CD68. G) Representative images of immunohistochemistry staining of ASH1L and CD68 in HCC TMA (cohort 2). Scale bars, 200 µm (left panel); 100 µm (right panel). H) EPIC analysis revealed a positive correlation between *ASH1L* expression and cancer‐associated fibroblast from the TCGA‐LIHC dataset. ANTISEQ analysis revealed a positive correlation between *ASH1L* expression and M2 macrophage infiltration from the TCGA‐LIHC dataset. Data are presented as mean ± SD. *P* values were computed using the paired Student's *t*‐test (A), Pearson correlation analysis (B,C), log‐rank test (D–F). ^∗∗^ *P* < 0.01, ^∗∗∗^ *P* < 0.001.

Finally, we examined the significance of ASH1L using clinical samples. *ASH1L* expression was significantly higher in tumors than adjacent nontumor tissues in our in‐house HCC cohort (cohort 1, 99 pairs), consistent with results observed from TCGA (**Figure** **8**A; Figure S9A, Supporting Information). To further differentiate the expression of ASH1L in hepatoma cells and HSCs, IHC analysis was performed on a tissue microarray (TMA) containing 196 cases of HCC primary tumor tissues (cohort 2). Positive staining of ASH1L was observed in both hepatoma cells and nonparenchymal cells, with universal strong staining in tumor cells and differential staining in mesenchymal cells. Statistical analysis revealed that high expression of ASH1L protein in mesenchyma was associated with poor histological differentiation (*P*< 0.01) and M2 macrophage marker CD68 expression (*P*< 0.01) (Figure 8B,C,G). A significant correlation between high *ASH1L* mRNA expression and advanced HCC stage was also found in the TCGA database (Figure S9B,D, Supporting Information). It is worth noting that patients with high expression of both ASH1L and CD68 have poorer overall survival and recurrence free survival rates compared to patients with high expression of ASH1L or CD68 alone (Figure 8D–F; Figure S9C, Supporting Information). In addition, TCGA data analysis showed that the expression of *ASH1L* was positively correlated with the infiltration of cancer‐associated fibroblast and M2 macrophage in HCC specimens, which was consistent with our observation in murine HCC model that ASH1L regulates HSC activation and macrophage polarization (Figure 8H). These data suggest that the expression of ASH1L in hepatocytes and HSCs is clinically associated with HCC progression and macrophage infiltration as well as polarization.

## 3 Discussion

H3K4 methyltransferases have been implicated in regulating gene expression during the development of various cell types and the occurrence of different diseases. To mitigate fibrosis or HCC, activated HSCs or malignant cells could be epigenetically reversed back to their quiescent or benign statuses. We screened out ASH1L, an H3K4 methyltransferase with higher expression levels in both hepatoma cells and activated HSCs, to avoid generating distinct effects when epigenetically targeting these two cell types. ASH1L regulates gene expression through methylation of lysine 4 and lysine 36 on histone H3, and mutations in it are clinically associated with autism spectrum disorder and intellectual disability.^[^ ^19^, ^23^, ^24^ ^]^ It has also been functionally linked to leukemogenesis and thyroid follicular tumorigenesis.^[^ ^25^, ^26^, ^27^ ^]^ Although ASH1L expression has been found to be up‐regulated during HSC activation and HCC development, its role in fibrosis‐associated HCC remains elusive.^[^ ^13^, ^28^, ^29^ ^]^ There is a notable absence of in vivo evidence, mainly due to the lack of appropriate models. *Ash1l* ^−/−^ mice are early embryonic lethal as ASH1L is essential for embryonic development.^[^ ^30^ ^]^ *Alb‐Cre* transgenic mouse and adeno‐associated virus (AAV)‐mediated gene delivery system exhibit specific tropism to hepatocytes while failing to target HSCs. In the present study, we used the *Gfap‐CreERT2* transgenic mouse to achieve temporal control of *Ash1l* knockout in HSCs upon tamoxifen administration. By crossing this HSC‐specific knockout mouse with the hepatocyte‐specific knockout mouse, we revealed that simultaneous depletion of *Ash1l* in both hepatocytes and HSCs mitigates fibrosis and HCC development.

Our results showed that double deletion of *Ash1l* inhibits the development of fibrosis‐associated HCC in vivo, whereas it has no effect on the proliferation of HCC cells in vitro. This suggests that ASH1L may function by modulating the tumor microenvironment. Sc‐RNA seq analysis showed that the proportion of pro‐tumorigenic TAMs was reduced, while the proportion of anti‐tumorigenic TAMs, T helper cells, and activated CD8^+^ T cells was increased in the DKO group compared to the WT group. TAMs are a major type of tumor‐infiltrating immune cells and are conventionally categorized into two polarized subtypes: M1 (anti‐tumor) and M2 (pro‐tumor).^[^ ^31^ ^]^ Recent data have revealed the complexity of TAMs in vivo. Traditional M1 and M2 markers are not mutually exclusive and additional markers such as CXCL9 and SPP1 have been added to better define M2‐like pro‐tumorigenic TAM subpopulations.^[^ ^14^ ^]^ For the first time, our findings provide a comprehensive profile of TAM subpopulations modulated by ASH1L in vivo. Our results also verified that ASH1L at least partially inhibits T cell‐mediated anti‐tumor response by recruiting and polarizing M2‐like TAMs. However, we cannot rule out the possibility that HSC‐ and hepatoma‐specific ASH1L might directly or indirectly modulate T cells via other pathways independent of M2‐like TAMs.

We then identified CCL2 and CSF1 as two critical downstream effectors of ASH1L to recruit and polarize M2‐like TAMs. These effectors have been extensively researched and their crucial roles in TAM recruitment and polarization have been well‐established.^[^ ^32^, ^33^ ^]^ We have detected CCL2 and CSF1 expression in both activated HSCs and hepatoma cells, which may explain why only a double knockout of ASH1L in both cell types, rather than a single knockout in either cell type, can alleviate the development of fibrosis‐associated HCC. Intriguingly, the expression levels of ASH1L, CCL2, and CSF1 further decreased in hepatoma cells if ASH1L was simultaneously knocked down in the cocultured HSCs, and vice versa (data not shown). This implies that hepatoma cells and HSCs may crosstalk with each other to strengthen the synergetic effect. Further research is needed to uncover the mechanisms through which these two cells communicate with each other. In addition, the levels of H3K4me3 modification in CCL2 and CSF1 promoters changed in accordance with ASH1L expression, suggesting that ASH1L regulates CCL2 and CSF1 expression by deposing these transcriptional activation markers on target gene promoters. It is possible that ASH1L works in coordination with specific transcription factors or other epigenetic modifications, such as H3K27me3, to regulate target gene expression. It remains to be seen what mechanisms govern the recruitment of ASH1L to these specific regions.

Epigenetic reprogramming of cells in the tumor milieu is crucial to the clinical implementation of efficient tumor management. However, identifying specific epigenetic regulators that are crucial for fibrosis‐related hepatocarcinogenesis remains one of the most challenging issues in this field. Our study concludes that hepatocyte‐specific and HSC‐specific ASH1L synergistically fosters an immunosuppressive microenvironment by recruiting and polarizing M2‐like macrophages via CCL2 and CSF1. ASH1L may be a small‐molecule therapeutic or immunotherapeutic target against fibrosis‐associated liver cancer.

## 4 Experimental Section

### Animal Experiments

The Institutional Animal Care and Use Committee at Southern University of Science and Technology reviewed and approved all animal experiments (SUSTC‐2019‐069). Male and female *C57BL/6J* mice were purchased from the GemPharmatech. *Ash1l ^flox/flox^* mice (Shanghai Model Organisms Center) were used as the control group (WT). Hepatocyte‐specific *ASH1L* knockout (HKO) mice were created by crossing *Ash1l ^flox/flox^* mice with *Albumin‐Cre* (*Alb‐Cre)* mice (Shanghai Model Organisms Center). HSC‐specific *ASH1L* knockout (SKO) mice were generated by crossing *Ash1l ^flox/flox^* mice with *Gfap‐CreERT2* (Shanghai Model Organisms Center) mice. Tamoxifen (75 mg kg^−1^ body weight, dissolved in corn oil) was intraperitoneally injected to 6‐week‐old mice for five consecutive days to induce *Ash1l* deletion in HSCs. Hepatocyte and HSC double deficient (DKO) mice were obtained by crossing *Ash1l ^flox/flox^* mice with *Alb‐Cre* and *Gfap‐CreERT2* mice.

### Mouse HCC Model

To induce the HCC model, a dose of 20 mg kg^−1^ diethylnitrosamine (DEN) (Sigma, # N0258‐1 g) was injected intraperitoneally 14 days after birth. Starting 8 weeks after birth, a weekly intraperitoneal injection of carbon tetrachloride (CCl_4_) (2 µL g^−1^ body weight, prediluted at 1:5 olive oil) was administered to promote HCC progression. The mice were sacrificed at different time points to monitor fibrosis and hepatocarcinogenesis.

### CCL2 Neutralizing Antibody and BLZ945 Mouse Therapeutic Model

C57BL/6J mice were induced to develop HCC using DEN‐CCl_4_. Beginning in week 18, the mice were randomly selected for treatment by intraperitoneal injection with CCL2‐neutralizing antibody (8 mg kg^−1^) (BioXcell, #BE0185) and oral gavage of BLZ945 (200 mg kg^−1^) (MCE, #HY‐10981A). Control groups were administered isotype IgG (BioXcell, #BE0091) and DMSO (Sigma, #D2650) in equivalent volumes. The treatments were administered four times weekly, the mice were sacrificed in week 24, and liver tissues were collected for imaging and statistical analysis.

### Hepa 1–6 Cell‐Induced Syngeneic Orthotopic Liver Cancer Model

Six‐ to eight‐week‐old male C57BL/6J mice were injected with 3.5 × 10^6^ Hepa1‐6 cells into the left liver lobe. Two weeks post‐inoculation, CCl_4_ (4 µL g^−1^ body weight, prediluted at 1:5 olive oil) was administered intraperitoneally every other day for three consecutive injections. Simultaneously, AS‐99 (30 mg kg^−1^) (MCE, #HY‐141429A) or DMSO control was administered intraperitoneally four times per week. Mice were sacrificed 35 days after tumor inoculation, and liver tissues were collected for imaging and statistical analysis.

### Cell Lines

The human HCC cell line CRL‐8024 was obtained from the Institute of Virology, Chinese Academy of Medical Sciences (Beijing, China). The normal human hepatocytes (CRL‐2706) and human HCC cell line SNU‐449 (CRL‐2234) were obtained from the American Type Culture Collection (ATCC). The human hepatic stellate cell line LX2 was obtained from Jennio Biotech (Guangzhou, China). Human embryonic kidney 293T cells were obtained from the National Collection of Authenticated Cell Cultures (Shanghai, China). Human monocytic cell line THP‐1 was a kind gift from Dr. Chao Liang (Southern University of Science and Technology, Shenzhen, China). Murine macrophage cell line Raw264.7 was a kind gift from Dr. Jun Wan (Southern University of Science and Technology, Shenzhen, China). Mouse epithelioid fibroblast cell line L929 was a kind gift from Dr. Li Zhang (Southern University of Science and Technology, Shenzhen, China). All cell lines were authenticated by short tandem repeat profiling and routinely tested for the absence of mycoplasma contamination. Cells were cultured in Dulbecco's modified Eagle's medium (DMEM) (Gibco, #C11995500BT) or RPMI‐1640 (Gibco, #C11875500BT), supplemented with 10% fetal bovine serum (Gibco, #10270106), and 1% penicillin/streptomycin mixture (Hyclone, #SV30010). All cell lines used in this study were incubated at 37 °C in a humidified incubator containing 5% CO_2_.

### Plasmids, Lentivirus Production, and Cell Infection

Two ASH1L knockdown vectors with pLL3.7 plasmid (Addgene) were constructed. Specific shRNA oligonucleotides targeting *ASH1L* were cloned into the pLL3.7 lentiviral vector. These plasmids and lentivirus packaging vectors from pLenti6/V5 Directional TOPO Expression Kit (Invitrogen) were co‐transfected into 293T cells. After 2 days, the viral particle supernatant was collected and transduced into cell lines. Stable knockdown cells were selected with puromycin (Gibco, #A1113803).

### Tet‐On System‐Mediated Knockdown

Specific shRNA oligonucleotides targeting *ASH1L* and the nonsense oligonucleotide were cloned into the pBX‐093 plasmid (PB5‐HS4‐SV40‐puro‐2A‐tetON3G‐pA‐HS4‐TRE‐AzaminGreen‐2A‐Tet3G‐RNAiGpA‐HS4‐PB3, a gift from Dr. Wei Huang, Southern University of Science and Technology, Shenzhen, China) respectively. These plasmids and transposase pBX‐090 plasmid (pN1‐CMV‐PGK‐piggybac, a gift from Dr. Wei Huang) were co‐transfected into HCC cells. Stable knockdown cells were selected with puromycin. Before the functional assays, cells were treated with 3 µg mL^−1^ doxycycline (DOX) (MCE, #HY‐N0565B) to induce the knockdown of ASH1L. Flow sorting based on green fluorescence was used to improve the knockdown efficiency of ASH1L.

### Cell Viability Assay, Foci Formation Assay, and Chemotactic Migration Assay

CCK‐8 assay was used to detect the cell viability. Cells were plated in 96 well plates at 1 × 10^3^ cells/well with 100 µL medium. After cell attachment, 10 µL CCK‐8 reagent (MCE, #HY‐K0301) was added to each well 3.5 h before analysis, and OD450 was detected for cell relative viability.

For the foci formation assay, cells were plated into 24 well plates at a density of 3 × 10^2^ cells/well with 1 mL of medium. After cell attachment, the medium was replaced with conditioned medium from macrophages (diluted 1:3). Two weeks later, the cells were fixed with 4% paraformaldehyde for 15 min and dyed purple with gentian violet. The number of foci was determined using the Image J software.

A chemotactic migration assay was conducted to detect the macrophage recruitment capacity. THP‐1 cells were pretreated with 150 ng mL^−1^ PMA (MCE, #HY‐18739) for 2 days and then were added to the upper chamber of a 24‐well transwell (8 µm pores, Falcon, #353097) for 2 h to attach to the membrane. The transwell chambers were then transferred to 24‐well plates containing 600 µL conditioned medium (diluted 1:3) from coculture systems and were incubated at 37 °C. After 2 days, the cells were fixed with 4% paraformaldehyde for 15 min and dyed purple with gentian violet. Image J software was used to determine the number of cells migrating to the membrane's other side.

### Cell Isolation

Primary hepatocytes and HSCs were isolated from the liver of normal C56BL/6 mice by density gradient centrifugation. Liver cells were isolated using two steps of digestion. The perfusion cannula was introduced into the inferior vena cava. The liver was perfused with washing solution (37 °C 50 mL D‐Hanks solution) for 5 min and then with the digestion solution (37 °C 20 mL collagen I + IV = 1:1, Sangon Biotech #A004194‐0100, #A004186‐0100. 0.2 mg mL^−1^ in D‐Hanks and DNase type I) for 5 min. The liver was carefully extracted and manually disrupted with a scalpel on a petri dish in a 2 mL digestion solution for 10 min. The digested liver was collected in 25 mL of PBS. Nondigested liver tissue was removed using a 40 µm Corning cell strainer (Sigma, #CLS431750). The liver was then washed with 1× PBS and treated with red blood cell lysis buffer to remove the red blood cells. The hepatocytes were collected by centrifuging for 3 min at 50 × *g* while the HSCs were found in the supernatant. To isolate the HSCs, 4 mL of 50% percoll (Solarbio, #P8370‐100ML), 2 mL of 35% percoll, and 2 mL of 20% percoll were added to a 15 mL centrifuge tube sequentially. The cell suspension was then added to the last layer and was centrifugated at 600 × *g* for 25 min. After centrifugation, a white material was left behind in the 20% percoll layer, which was the HSCs layer.

### Coculture Assay

The 6‐well transwell chambers (0.4 µm pores, Corning,# 3412) were used for the coculture assay. A total of 4 × 10^5^ LX2 cells or murine primary HSCs were added to the lower transwell chamber. After cell attachment, HCC cells or murine primary hepatocytes were seeded in the upper chamber of the coculture system for 2 days. The conditioned medium was collected and centrifuged for 10 min at 2000 × *g* to remove cell precipitates.

### Culture of Bone Marrow‐Derived Macrophages (BMDMφ)

The conditioned medium (CM) of L929 cells was collected and filtered through a 0.45 µm filter as the differentiation medium (prediluted at 1:4 DMEM complete medium). Bone marrow (BM) was harvested from *C57BL/6* mice and cultured in the differentiation medium for 7 days to induce the differentiation of M0 macrophages. Supplement cells were given an additional fresh differentiation medium on day 3, and the new differentiation medium was replaced on day 5.

### In Vitro T Cell Assays

Murine CD8^+^ T cells were purified from spleens of *C57BL/6* OT‐1 transgenic mice using an immunomagnetic system (Thermofish, #11413D8804‐6822‐74), and the cell purity was typically>90%. For the carboxyfluorescein diacetate succinimidyl ester (CFSE) assay, the CD8^+^ T cells were labeled with CFSE (Biolegend, #423801) and then stimulated with specific OVA (257‐26) (SIINFEKL) peptide (MCE, #HY‐P1489A). The resulting cells were analyzed using flow cytometry.

### Coculture of Macrophage with CD8+ T Cells

Bone marrow‐derived macrophages (BMDMφ) were incubated with a specific OVA (257‐264) (SIINFEKL) peptide at a concentration of 10 nM for 4 h. After washing with PBS buffer, the BMDMφ were cocultured with CFSE‐labeled CD8^+^T cells at a 1:2 ratio for 48 h. Following coculture, the CD8^+^ T cells were analyzed using flow cytometry.

### RNA Extraction, Reverse Transcription, and Quantitative Real‐Time PCR

According to the manufacturer's instructions, Trizol reagent was used for total RNA extraction in cells and tissues. 1 µg of total RNA was used to synthesize the first strand of cDNA using Hifair^®^ III 1st Strand cDNA Synthesis SuperMix (Yeasen, Shanghai, China, #11141ES60). The PCR product was analyzed by electrophoresis in 1% agarose gel. RT‐qPCR was performed using the SYBR^®^ Green Premix Pro Taq HS qPCR Kit (Accurate Biology, Changsha, China, #AG11701) according to the manufacturer's instructions. The primers used for RT‐qPCR are listed in Supplementary Table S1. The relative expression was calculated using the 2 ^−ΔΔCt^ method.

### RNA‐Seq Sample Preparation and Analysis

Bulk liver RNA sequencing was performed by Novogene (Beijing, China). Total RNA was extracted using Trizol reagent (Sigma, # T9424‐100ML), and mRNA was enriched with magnetic beads with Oligo (dT). RNA‐seq libraries were prepared after rRNA removal and sequenced using the Illumina platform. Paired‐end reads were mapped to the Homo sapiens GRCh38 (hg38) reference genome using the STAR RNA‐seq aligner. FeatureCounts obtained raw read counts. Next, the DESeq2 R package for differential gene analysis (*P* ≤ 0.05 and fold change ≥ 2) was used. GO enrichment analysis was performed using the ClusterProfiler R package. The TPM value was used to assess the expression levels of genes for heatmap plotting.

### Single‐Cell Data Analysis

Mouse liver tissue digestion and single‐cell sequencing were performed by Novogene (Beijing, China). The raw single‐cell RNA‐seq data were processed using the 10X Genomics Cell Ranger toolkit (version 4.1.0), which includes reading alignment from FASTQ files to the human reference genome (GRCh38, v3.0.0, obtained from 10X Genomics) and generation of a gene‐cell unique molecular identifier (UMI) matrix using the “cell ranger count” function. Quality control was performed using the Seurat v5 R package. Several steps were taken to filter out low‐quality data. Cells with fewer than 200 or more than 5000 expressed genes and those containing fewer than 400 or more than 25000 UMIs were removed. Additionally, cells with mitochondrial gene expressions exceeding 10% were excluded.

After performing quality control and filtering, dimensionality reduction and annotation of the cells were performed. SCTransform function was performed to normalize data. Principal component analysis (PCA) was conducted to reduce the dimensionality of the data, followed by clustering of the cells based on the PCA results. Subsequently, Uniform Manifold Approximation and Projection (UMAP) was applied to visualize the high‐dimensional data in two dimensions.

### ChIP Analysis

The ChIP experiments were performed according to a previous protocol.^[^ ^34^ ^]^ Hepatoma cells and HSCs were cocultured for two days, and then 2 × 10^6^ cells from each cell line were fixed in 1% formaldehyde and resuspended in a sonication buffer. The chromatin DNA was sonicated and sheared to lengths ranging from 200 to 1000 bp. The sheared chromatin was immunoprecipitated with an anti‐H3K4me3 antibody (Active Motif, #39016) overnight at 4 °C. The DNA was then purified using a DNA Clean kit (ZYMO, #D4013).

The data analysis was processed as previously described:^[^ ^35^ ^]^ raw reads from fastq files were first trimmed using fastp (0.20.071) with the setting “‐l 25–detect_adapter_for_pe.” The trimmed reads were then mapped to hg38 by hisat2 (2.1.072) with default setting and sorted by samtools (1.973) with flag “‐ShuF 4 ‐q 30 ‐f 2.” After mapping, duplicated reads were removed by the Picard tool (v.2.20.8) (http://broadinstitute.github.io/picard/). Peaks were then called using MACS2 (2.1.474) with the setting “‐f BAMPE ‐g hs –keep‐dup all ‐B –SPMR ‐q 0.01.” MAnorm was used to identify differential peaks between different biological conditions with default parameters. The promoter region was defined as the 2000 bp before the TSS region. *P* ≤ 0.05 and fold change ≥1.5 were screening criteria to find the target genes corresponding to the differential sites with more than 50% peak coverage of the promoter region.

ChIP‐qPCR was used to detect the bound regions related to genes of interest, and the primers used were listed in Supplementary Table S1.

### Flow Cytometry Assay

Single‐cell suspensions were stained with fluorochrome‐conjugated antibody proteins (Supplementary Table S2). Fluorochrome‐conjugated antibodies were used for direct or indirect staining. These samples were analyzed using a Beckman Cytoflex Analyzer. Data analysis was performed using FlowJo software.

### Western Blot

The liver tissues or cells were resuspended in RIPA lysis buffer (Sigma, # R0278‐50 mL) for 20 min on ice. After centrifugation at 12 000 × *g* for 15 min, an equal amount of proteins was boiled in loading buffer, separated on 10% SDS–polyacrylamide gel (Yeasen, #20325ES62) electrophoresis, and electrotransferred to polyvinylidene difluoride membranes (Merck, #IPVH00010). The membranes were blocked for an hour with 5% bovine serum albumin (Solarbio, #A8020). After incubation with specific antibodies against α‐SMA (eBioscience, #14‐9760‐80), H3K4me3 (ActiveMotif, #3916), H3K36me3 (Active Motif, #61902), ASH1L (Novus, #NB10093290), CCL2 (Abcam, #ab7202), CSF1 (Abcam, #ab233387), GAPDH (CST, #ab21188s), the presence of indicated protein on the blots were detected with a commercial ECL kit (Bio‐rad, #170‐5061).

### Immunohistochemistry

Paraffin‐embedded and formalin‐fixed samples were cut into 5‐µm sections, followed by procedures for immunohistochemistry. After incubation with primary antibody against ASH1L (1:50, Bethyl, #A301‐749A‐T), CD68 (1:500, Proteintech, #28058‐1‐AP), ARG1 (1:100, CST, #93668T), CCL2 (1:300, Abcam, #ab7202) and CSF1 (1:500, Abcam, #ab233387) overnight at 4 °C, sections were stained with polyperoxidase‐anti‐rabbit IgG (Boster, #SV0002) and DAB Detection Kit (Biosharp, #BL732A). After being counterstained with Harris hematoxylin (Solarbio, #G4070), dehydrated with graded alcohols, bathed in fresh xylene, and covered with gummi, the sections were visualized in a pathological section scanning system (NanoZoomer S60).

A tissue microarray containing 196 primary HCC tumor tissues (cohort 2) was used for the immunohistochemical detection of ASH1L and CD68 expression. ASH1L staining was detected in 123 cases, and CD68 staining was detected in 188 cases. The expression levels of ASH1L and CD68 in HCC were scored as the proportion of the immune‐positive staining area (0‒100%) multiplied by the intensity of staining (0, negative; 1, weak; 2, moderate; and 3, intense). Dr. Shaoyan Xi determined the scores. The median IHC score was chosen as the cutoff value for defining high and low expression of ASH1L.

### ELISA

ELISA was carried out following the manufacturer's instructions. Briefly, the conditioned medium from the coculture system was centrifuged for 10 min at 2000 × *g* to remove any floating cells. The secreted CCL2 and CSF1 were quantified using the human CCL2/MCP‐1 Quantikine ELISA Kit (Raybiotech, #ELH‐MCP1‐1), human M‐CSF Quantikine ELISA Kit (Raybiotech, #ELH‐MCSF‐1), mouse CCL2/MCP‐1 ELISA Kit (LIANKE, #70‐EK287/2‐96), and mouse M‐CSF ELISA Kit (LIANKE,#70‐EK2144‐96).

### TCGA Data Analysis

The HCC transcriptome data were obtained from The Cancer Genome Atlas Liver Hepatocellular Carcinoma (TCGA_LIHC) project. The differences in ASH1L expression and survival were analyzed by SPSS 24.0 or GraphPad Prism 8.0. The expression of ASH1L at different tumor stages was obtained online via UALCAN (ualcan.path.uab.edu/analysis.htmL). Correlation analysis of ASH1L expression and infiltration of cancer‐associated fibroblast and M2 macrophage in HCC was obtained online via TIMER2.0 (TIMER2.0 (cistrome.org).

### Clinical Sample

A total of 99 pairs of frozen primary HCC tumor tissues and adjacent nontumor tissues (cohort 1) were collected with informed consent from patients who underwent hepatectomy at Sun Yat‐sen University Cancer Center (Guangzhou, China). RT‐qPCR was used to detect the mRNA expression of *ASH1L* in cohort 1 patients. A tissue microarray (TMA) containing 196 primary HCC tumor tissues (cohort 2) was used for the immunohistochemical detection of protein expression. Ethics Committee of Sun Yat‐sen University Cancer Center approved the clinical specimens used in this study (B2024‐255‐01).

### Statistical Analysis

SPSS 24.0 or GraphPad Prism 8.0. was used for statistical analysis. Paired two‐tailed Student's *t*‐test was used to analyze the flow cytometry experiments performed in batches, and the mRNA level of *ASH1L* in paired clinical samples. Other expression analysis statistics were compared using unpaired two‐tailed Student's *t*‐test. All data are presented as the mean ± standard deviation (SD), *P* ≤ 0.05 was considered statistically significant. Statistical significance is denoted ^(∗^ *P* < 0.05, ^∗∗^ *P* < 0.01, ^∗∗∗^ *P *< 0.001, ^∗∗∗∗^ *P* < 0.0001, ns. not significant) in the figures and figure legends. Overall and recurrence‐free survival differences were calculated using Kaplan–Meier plots and log‐rank tests. The correlations between clinical parameters and the expression of ASH1L were analyzed using Pearson's correlation analysis. All experiments were independently repeated at least three times.

## Conflict of Interest

The authors declare no conflict of interest.

## Author Contributions

Y. Du, S. Wu, Y. Li – study concept/design; Y. Du, S. Wu, S. Xi, W. Xu, L. Sun, J. Yan, H. Gao, Y. Wang, J. Zheng, F. Wang, H. Yang – experimental data acquisition; Y. Du, S. Wu, W. Xu, J. Yan, Y. Li – analysis and interpretation of data; S. Xi, D. Xie – provided human samples and clinical data analyses; X. Chen, X. Ou, XY. Guan – provided valuable comments; Y. Du, S. Wu, Y. Li – drafted the manuscript; Y. Li – obtained funding, editing, and supervision; all authors have read and edited the manuscript.

## Supporting information



Supporting Information

## Data Availability

The data that support the findings of this study are available from the corresponding author upon reasonable request.
