# Supplementary material for: ASH1L in Hepatoma Cells and Hepatic Stellate Cells Promotes Fibrosis‐Associated Hepatocellular Carcinoma by Modulating Tumor‐Associated Macrophages
Source: Adv Sci (Weinh). 2024 Oct 8;11(45):2404756. doi: 10.1002/advs.202404756 (PMC11615825; doi:10.1002/advs.202404756)
Supplement: Supplementary file 1 — Supporting Information [file ADVS-11-2404756-s001.docx]

Supporting Information

**ASH1L in Hepatoma Cells and Hepatic Stellate Cells Promotes Fibrosis-associated Hepatocellular Carcinoma by Modulating Tumor-associated Macrophages**

*Yuyang Du, ^#^ Shasha Wu, ^#^ Shaoyan Xi, Wei Xu, Liangzhan Sun, Jingsong Yan, Han Gao, Yanchen Wang**, Jingyi Zheng, Fenfen Wang, Hui Yang, Dan Xie, Xi Chen, Xijun Ou, Xin-Yuan Guan, Yan Li**

*Correspondence: [liyan181@smu.edu.cn](mailto:liyan181@smu.edu.cn)

^#^equal contribution

Y. Du, S. Wu, L. Sun, J. Yan, H. Gao, F. Wang, H. Yang, X. Chen, Y. Li

Department of Systems Biology

School of Life Sciences

Southern University of Science and Technology

Shenzhen 518055, China

S. Xi

Department of Pathology

Sun Yat-Sen University Cancer Center

Guangzhou 510275, China

S. Xi, D. Xie, XY. Guan

State Key Laboratory of Oncology in South China and Collaborative Innovation Center for Cancer Medicine

Sun Yat-sen University Cancer Center

Guangzhou 510080, China

W. Xu

GMU-GIBH Joint School of Life Sciences

The Guangdong-Hong Kong-Macau Joint Laboratory for Cell Fate Regulation and Diseases

Guangzhou Medical University

Guangzhou 511436, China

L. Sun, XY. Guan

Department of Clinical Oncology

The University of Hong Kong

Hong Kong 999077, China

L. Sun

Institute of Cancer Research

Shenzhen Bay Laboratory

Shenzhen 518067, China

Y. Wang, J. Zheng, Y. Li

Shenzhen Hospital

Southern Medical University

Shenzhen 518000, China

E-mail: liyan181@smu.edu.cn

X. Ou, Y. Li

School of Life Sciences

Southern University of Science and Technology

Shenzhen 518055, China

XY. Guan

The University of Hong Kong-Shenzhen Hospital

Shenzhen 518053, China

# Supporting Figures

**
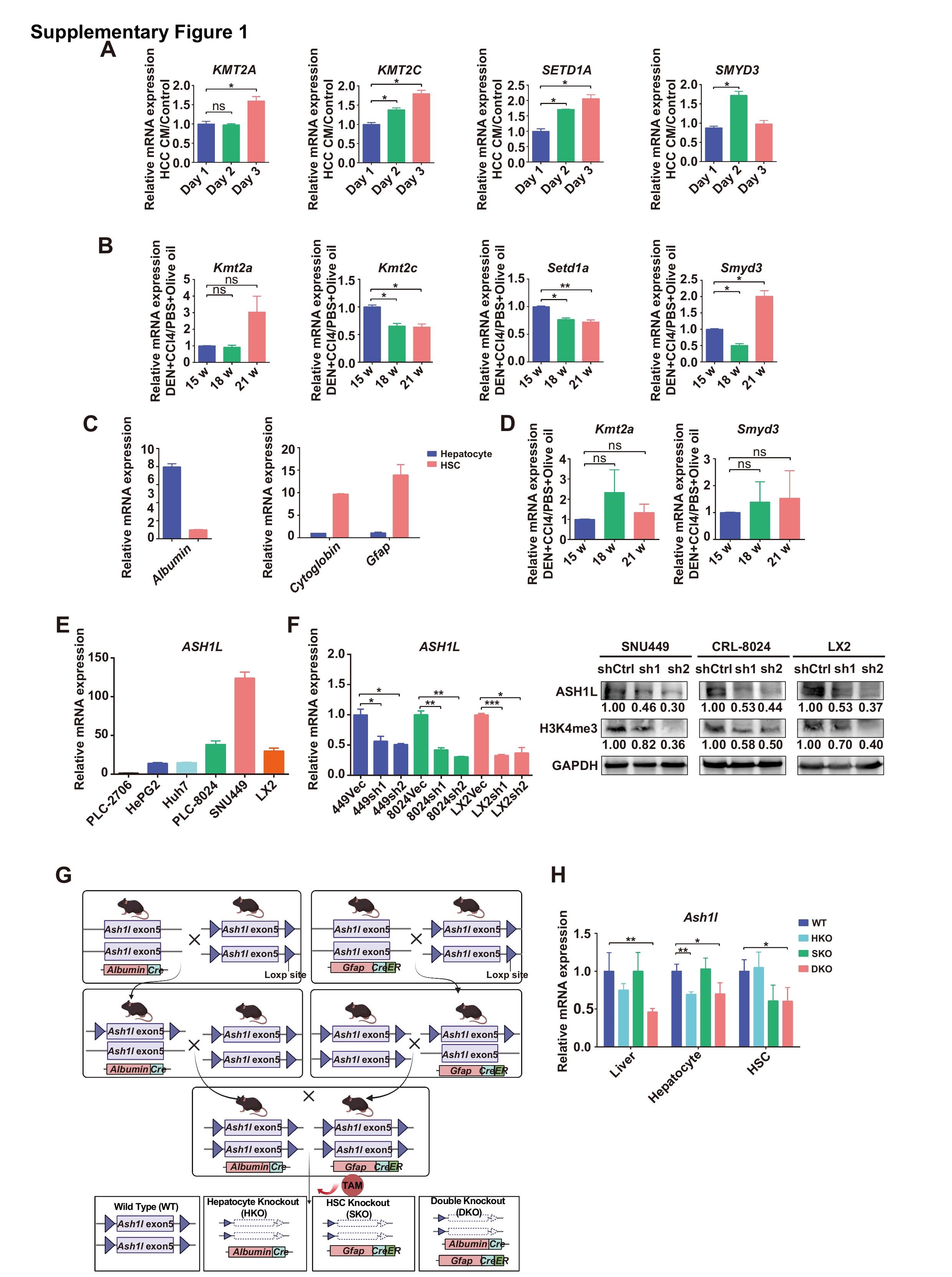
**

**Figure S1. Construction of ASH1L knockdown stable cell lines and transgenic mice.**

(A) RT-qPCR analysis assessed the relative mRNA expression of H3K4me3 methyltransferases in LX2 cells after HCC CM treatment. (B) RT-qPCR analysis assessed the relative mRNA expression of H3K4me3 methyltransferases in the DEN / CCl_4_-induced HCC tissues compared with the DMSO + Olive oil control treatment. (C) Relative mRNA expression of the hepatocyte marker gene (*Albumin*) and HSC marker genes (*Cytoglobin, Gfap*) in hepatocytes and HSCs isolated by density gradient centrifugation (*n*=2). (D) Expression of H3K4me3 methyltransferases in the HSCs of DEN/CCl_4_-induced HCC mice. (E) Expression of *ASH1L* in normal human hepatocytes (PLC-2706) and HCC cell lines and HSC cell line (LX2). (F) RT-qPCR (left) and western blots (right) analysis results confirmed the knockdown efficiency of ASH1L. (G) Breeding schemes for transgenic mice. (H) RT-qPCR analysis showed the *Ash1l* mRNA expression in different transgenic mouse groups' total livers, hepatocytes, and HSCs (*n*=3). Data are presented as mean ± SD. *P* values were computed using the unpaired Student’s t-test (A, B, D, F, H). ∗*P* < 0.05, ∗∗*P* < 0.01, ∗∗∗*P*< 0.001. ns. not significant.


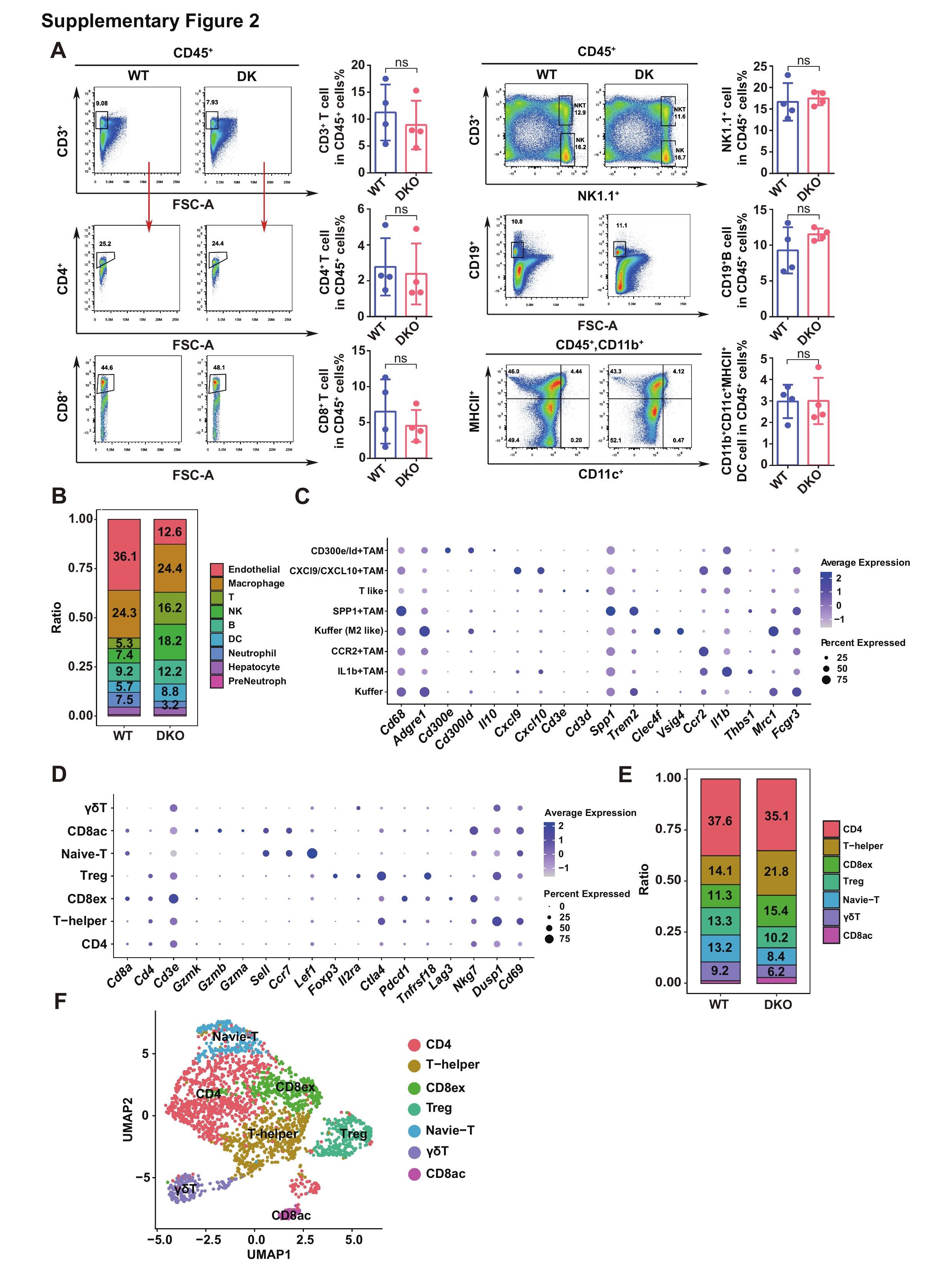


**Figure S2. ASH1L expression in hepatocytes and HSCs modulates immune micro-environment in fibrosis-associated HCC.**

(A) The proportion of different immune cells in the liver tumor tissues of WT and DKO mice was detected using multicolor flow cytometry (*n*=4). (B) Percentage of different cells in WT and DKO mice. (C, D) Bubble heatmap depicting the expression levels of cluster-specific marker genes in macrophage (C) or T cell (D) subpopulations. (E) Percentage of different T cell subclusters in WT and DKO mice. (F) UMAP representation of T cell subclusters. Data are presented as mean ± SD. *P* values were computed using the paired Student’s t-test (A). ns. not significant.


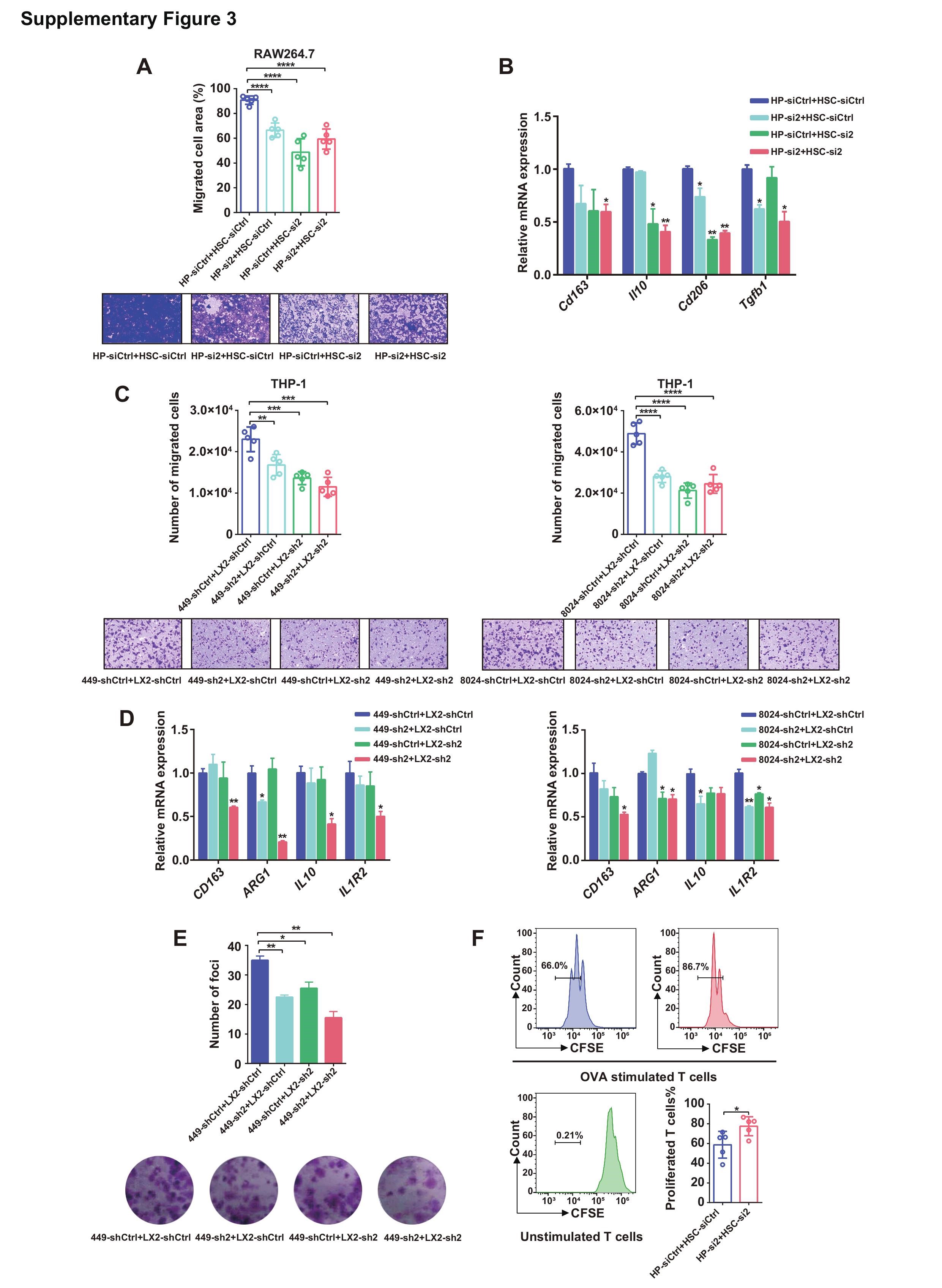


**Figure S3. Knockdown of ASH1L in hepatocytes and HSCs impairs macrophage recruitment and M2 polarization.**

(A) Chemotactic migration assays of Raw264.7 mouse macrophage cell line using CM from the indicated mouse primary hepatocytes (HP) and HSCs (*n*=5). (B) RT-qPCR analysis evaluated the expression of the M2 markers in BMDMs that were treated with CM of co-cultured mouse primary hepatocytes and HSCs. (C) Human monocyte line THP-1 was induced to M0 macrophages with PMA (150 ng/ml) treatment for 48 h. Chemotactic migration assays of macrophages using the CM of co-cultured human HCC cell lines SNU449 /CRL-8024 and HSC cell line LX2 as indicated (*n*=5). (D) RT-qPCR assessed the expression of the M2 markers of macrophages treated with the indicated CM. (E) Representative images and quantification of tumor cell foci when cultured with CM from polarized macrophages induced by the co-cultured SNU449 and LX2 cells as indicated (*n*=3). (F) CFSE histograms detected the proliferation of CD8^+^ T cells co-cultured with macrophages induced by CM of the indicated primary hepatocytes and HSCs (*n*=5). Data are presented as mean ± SD. *P* values were computed using the unpaired Student’s t-test (A-F). ∗*P* < 0.05, ∗∗*P* < 0.01, ∗∗∗*P*< 0.001, ∗∗∗∗*P* < 0.0001.


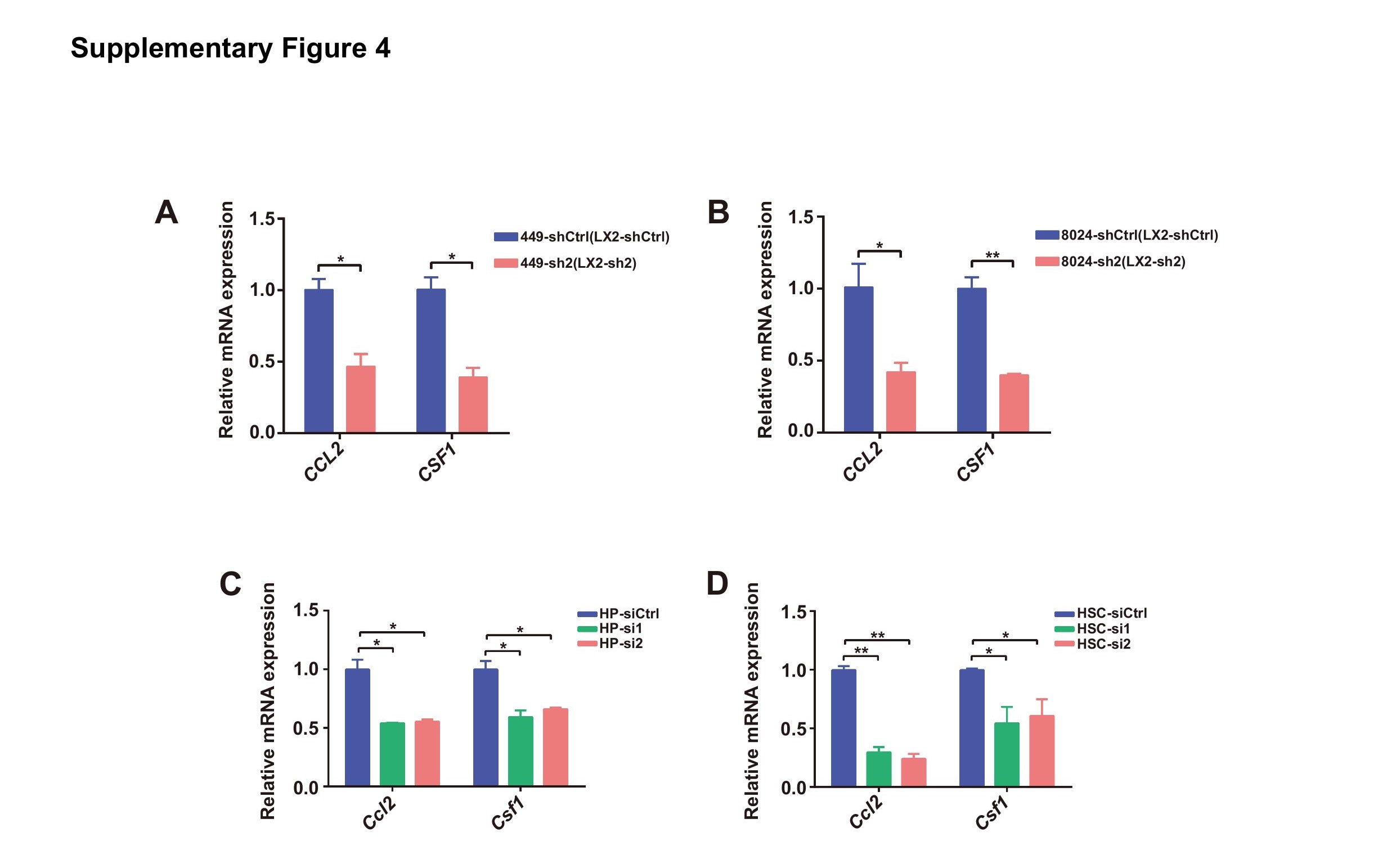


**Figure S4. Knockdown of ASH1L in hepatocytes and HSCs reduces CCL2 and CSF1 expression.**

(A, B) Relative mRNA expression of *CCL2* and *CSF1* in ASH1L knockdown HCC cell lines SNU449 (A) and CRL-8024 (B). (LX2-shCtrl): co-cultured with control LX2 cells, (LX2-sh2): co-cultured with sh2-mediated ASH1L knockdown LX2 cells. (C, D) Two siRNA targeting different CDS regions of *Ash1l* were transfected into murine primary hepatocytes (C) or HSCs (D). RNA was extracted 48 hours later, and RT-qPCR was used to measure the relative mRNA expression of *Ccl2* and *Csf1*. Data are presented as mean ± SD. *P* values were computed using the unpaired Student’s t-test (A-D). ∗*P* < 0.05, ∗∗*P* < 0.01.


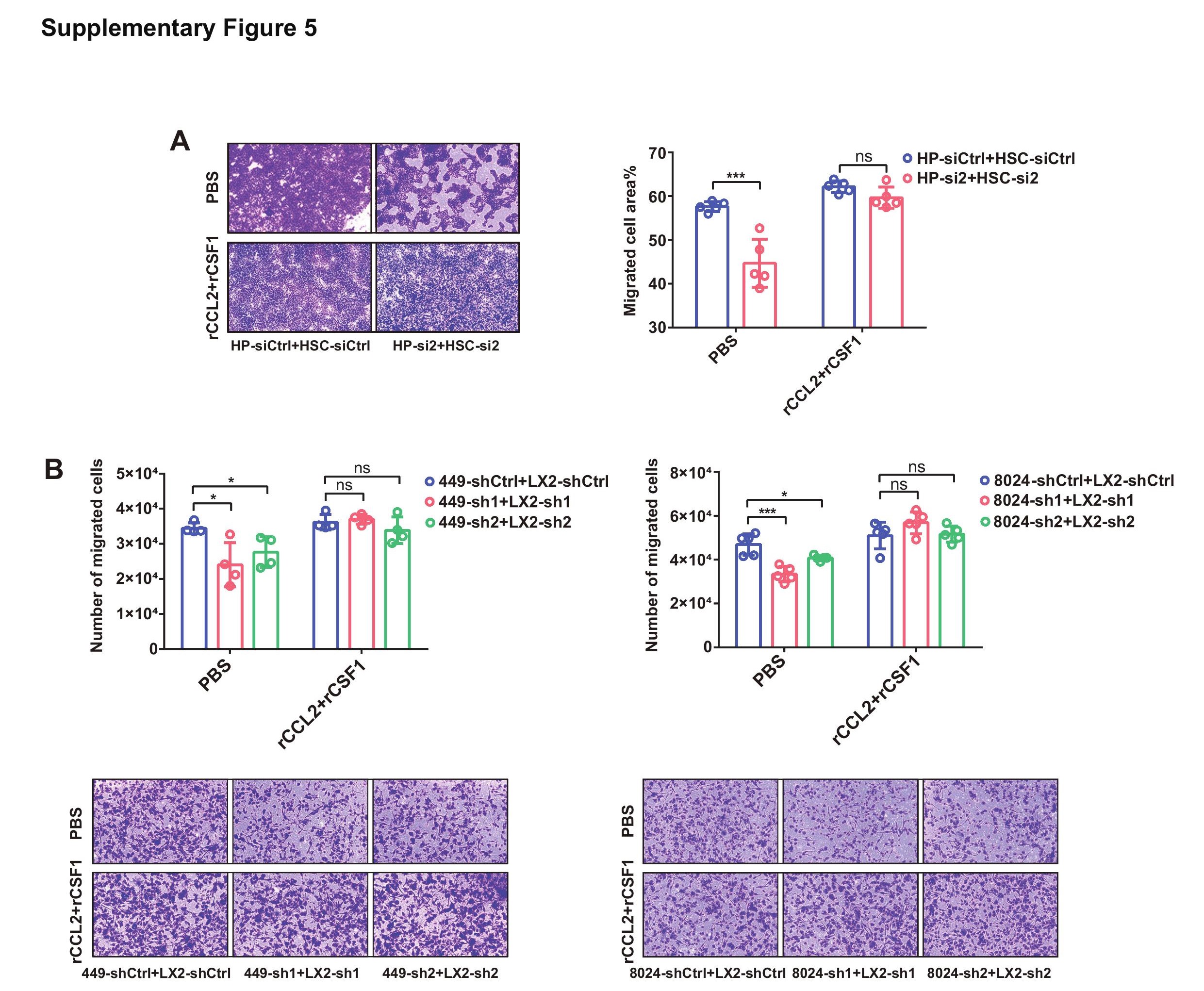


**Figure S5. Rescue of impaired macrophage recruitment by CCL2 and CSF1 recombinant proteins following ASH1L knockdown.**

(A) Chemotactic migration assays of macrophages using the indicated CM of co-cultured murine primary hepatocytes. ASH1L CDS area was targeted by si-ASH1L#2 (*n*=5). (B) Chemotactic migration assays of macrophages using the indicated CM of co-cultured human HCC cell line SNU449/CRL-8024 and LX2 cells. The conditioned media was supplemented with either PBS or 100 ng/mL of human recombinant protein rCCL2 and rCSF1 (rCCL2 + rCSF1) (*n*=5). Data are presented as mean ± SD. *P* values were computed using the unpaired Student’s t-test (A-B). ∗*P* < 0.05, ∗∗∗*P* < 0.001. ns. not significant.

**
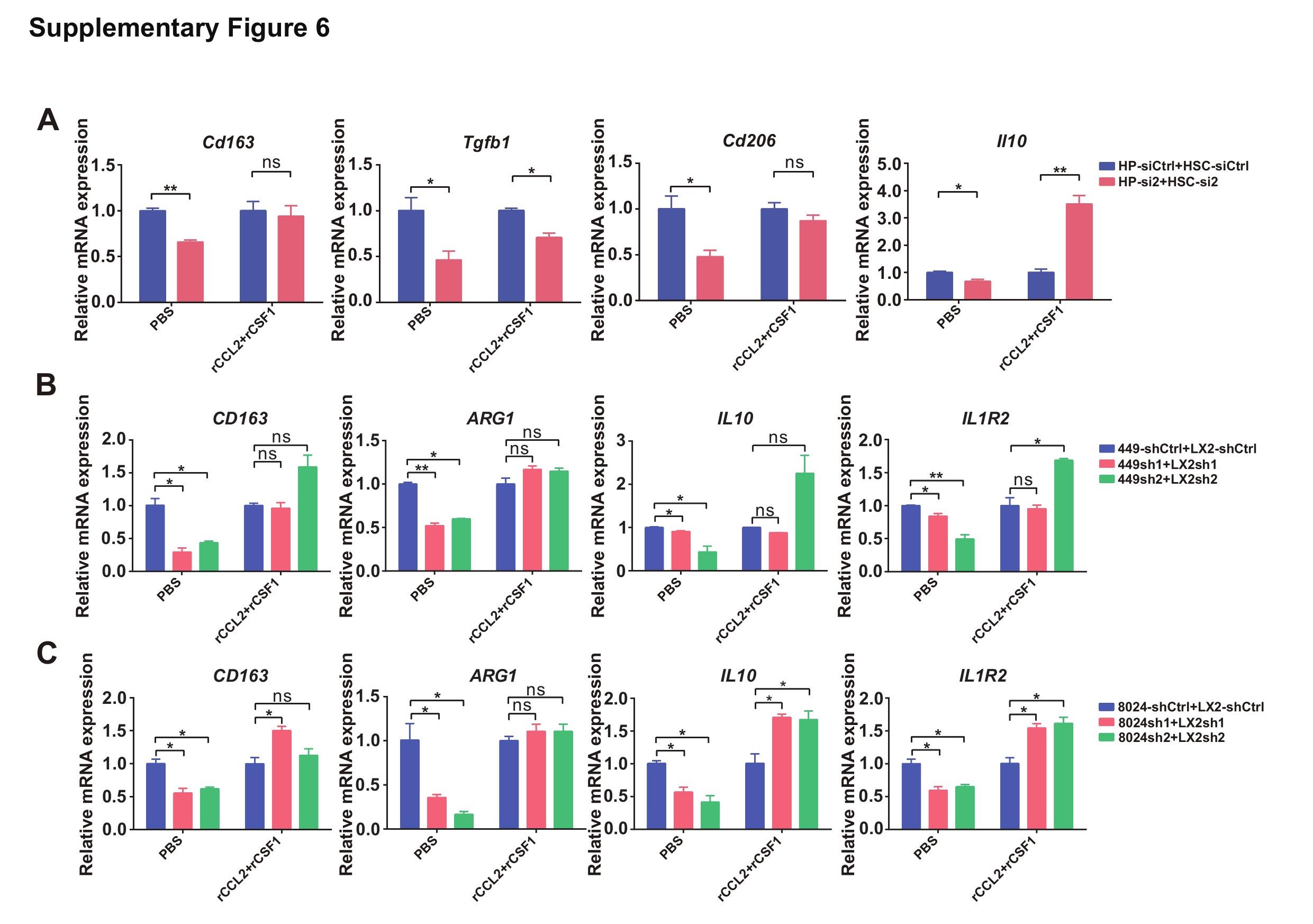
**

**Figure S6. Rescue of impaired macrophage M2-polarization by CCL2 and CSF1 recombinant proteins following ASH1L knockdown.**

(A-C) RT-qPCR analysis measured the relative mRNA expression of M2 markers in macrophages treated with the CM of co-cultured primary hepatocytes (HP) and HSCs (A), or co-cultured SNU449 and LX2 cells (B), or co-cultured CRL-8024 and LX2 cells (C). The conditioned media was supplemented with either PBS or 50 ng/mL of mouse recombinant proteins rCCL2 and rCSF1 (A), or 100 ng/mL of human recombinant protein rCCL2 and rCSF1 (B-C). ASH1L CDS area was targeted by si-ASH1L#2 or sh-ASH1L#1 or sh-ASH1L#2. Data are presented as mean ± SD. *P* values were computed using the unpaired Student’s t-test (A-C). ∗*P* < 0.05, ∗∗*P* < 0.01. ns. not significant.

**
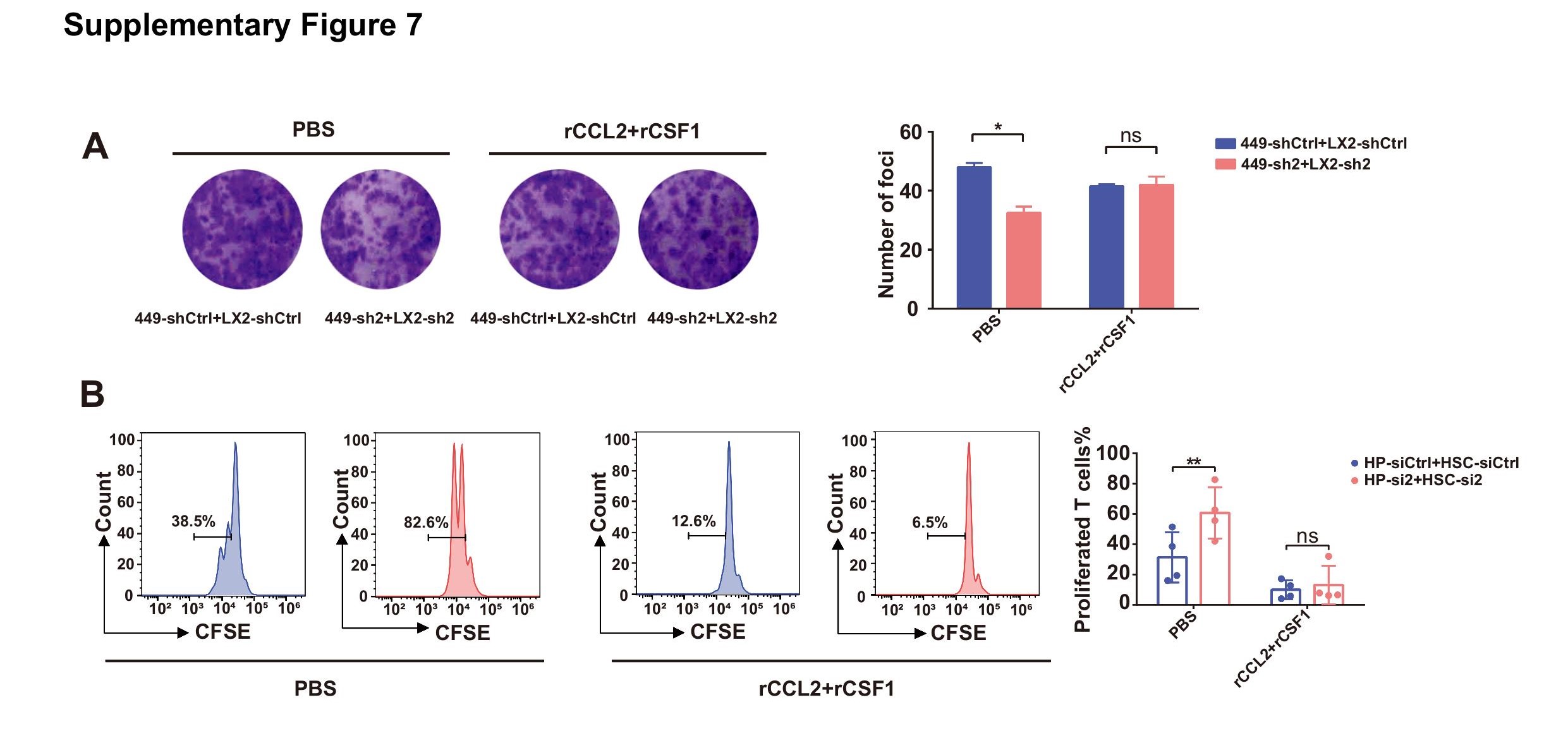
**

**Figure S7. Recombinant proteins of CCL2 and CSF1 reverse the inhibition of HCC cell proliferation and CD8+ T cell activation phenotypes caused by ASH1L knockdown.**

(A) Representative images and quantification of tumor cell foci when cultured with the indicated CM from polarized macrophages induced by the co-cultured SNU449 and LX2 cells. The conditioned media was supplemented with either PBS or 100 ng/mL of human recombinant protein rCCL2 and rCSF1 (*n*=3). (B) CFSE histograms detected the proliferation of CD8^+^ T cells co-cultured with macrophages induced by the indicated CM of primary hepatocytes and HSCs. The conditioned media was supplemented with either PBS or 50 ng/mL of mouse recombinant proteins rCCL2 and rCSF1. ASH1L CDS area was targeted by si-ASH1L#2 (*n*=4). Data are presented as mean ± SD. *P* values were computed using the unpaired Student’s t-test (A-B). ∗*P* < 0.05, ∗∗*P* < 0.01. ns. not significant.


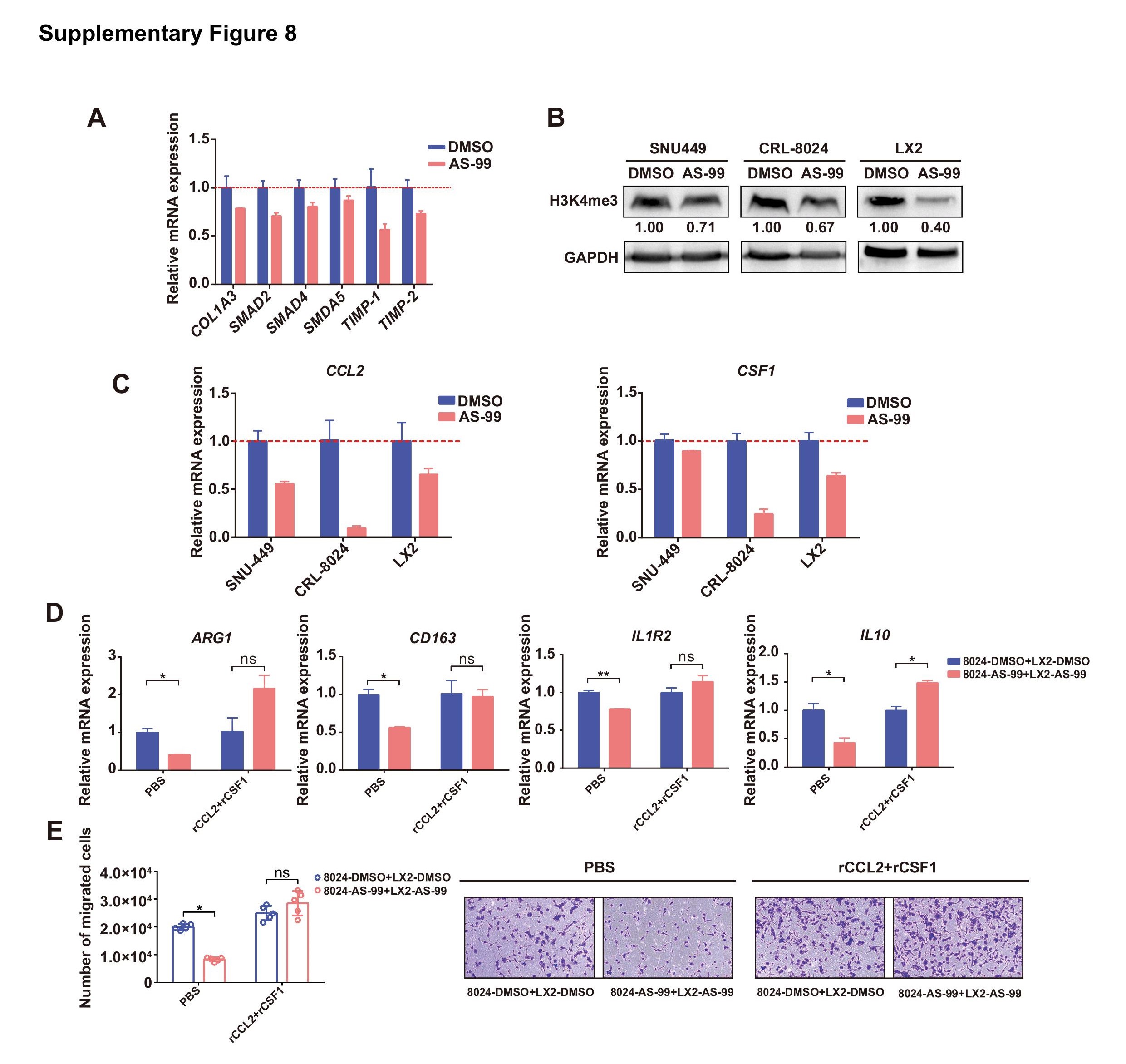


**Figure S8. AS-99 inhibits HSC activation and reduces the expression of *CCL2* and *CSF1.***

(A) RT-qPCR analysis measured the relative mRNA expression of HSC activation marker genes in LX2 after DMSO or AS-99 treatment. (B) DMSO- or AS-99- treated HCC and LX2 cells were cultured for 3 days, and the H3K4me3 modification level was measured using western blots. (C) DMSO- or AS-99- treated HCC and LX2 cells were cultured for 7 days, and the expression of *CCL2* and *CSF1* were measured using RT-qPCR. (D) RT-qPCR detected expression of M2 markers in macrophages treated with the CM from co-cultured CRL-8024 and LX2 cells. (E) Chemotactic migration assays of macrophages using the indicated CM of co-cultured CRL-8024 and LX2 (*n*=5). Data are presented as mean ± SD. *P* values were computed using the unpaired Student’s t-test (D-E). ∗*P* < 0.05, ∗∗*P* < 0.01. ns. not significant.


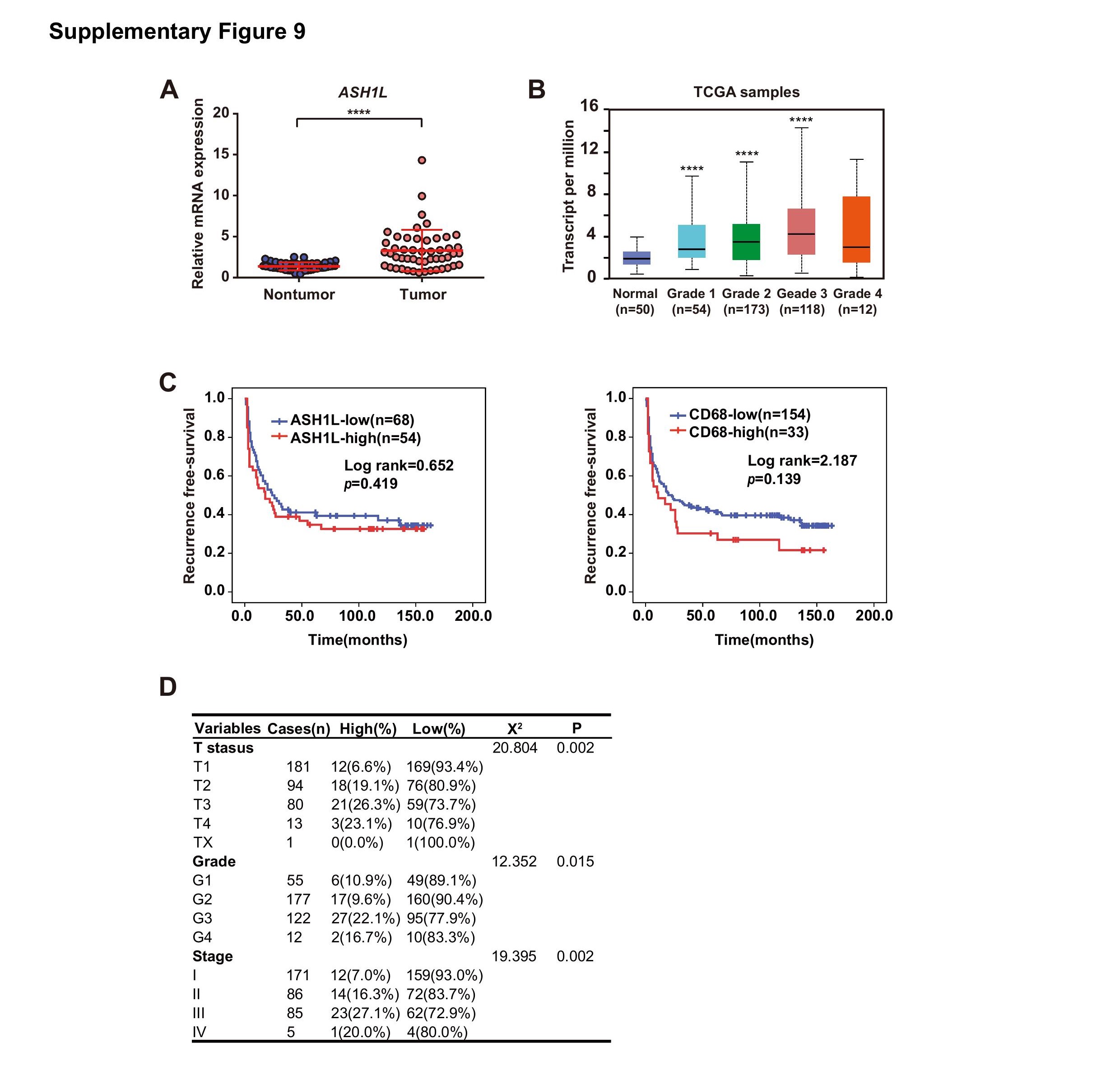


**Figure S9. Clinical significance of ASH1L in human HCC.**

(A) Normalized expression (FPKM) of *ASH1L* in HCC nontumor and tumor tissues from the TCGA database (*n*=50). (B) Expression of ASH1L in different grades of HCC from the TCGA-LIHC dataset. (C) Kaplan–Meier recurrence-free survival curves of HCC patients with normal or overexpressed ASH1L (left) and overexpressed CD68 (right).(D) Correlation analysis of ASH1L expression and clinical stage of HCC. *P* values were computed using the unpaired Student’s t-test (B) and paired Student’s t-test (A), log-rank test (C), and Pearson correlation analysis (D). ∗∗∗∗*P* < 0.0001.

**Supplementary Table S1. Sequences of nucleotides**

| **Primer name** | **Sequences** | **Application** |
| --- | --- | --- |
| *COL1A1*  (Human) | F: CGGCTCCTGCTCCTCTT | qRT-PCR |
|  | R: GGGGCAGTTCTTGGTCTC |  |
| *SMAD3*  (Human) | F: CCATCTCCTACTACGAGCTGAA | qRT-PCR |
|  | R: CACTGCTGCATTCCTGTTGAC |  |
| *TIMP-1* | F: CCAGAAGTCAACCAGACCACC | qRT-PCR |
| (Human) | R: GGGCAGGATTCAGGCTATCTG |  |
| *TIMP-2*  (Human) | F: AGAGGATCCAGTATGAGATCAAGCAG | qRT-PCR |
|  | R: TGGTACCTGTGGTTCAGGCTCTTC |  |
| *KMT2A*  (Human) | F: AAGAGCAGGTAAACTCTCTCCTC | qRT-PCR |
|  | R: TTCCTCTCCGTCGTACAATTTG |  |
| *KMT2C*  (Human) | F: TGGGTTCACCTAGAGTGTGAC | qRT-PCR |
|  | R: CTGGCTGTAAACGATCCATCTC |  |
| *SETD1A*  (Human) | F: CAGTGGCGGAACTACAAGCTC | qRT-PCR |
|  | R: CATAGCGGTACACCTTCTGAGA |  |
| *SMYD3*  (Human) | F: CGCGTCGCCAAATACTGTAGT | qRT-PCR |
|  | R: CAAGAAGTCGAACGGAGTCTG |  |
| *ASH1L*  (Human) | F: ACACTGTCCTTCAAAACGAGAC | qRT-PCR |
|  | R: GAAGAGTAGATGGCGTTGCATTA |  |
| *CD163*  (Human) | F: TTGCACAGATATTTCAGTGCAG | qRT-PCR |
|  | R: ACTGCAATAAAGGATGACTGAC |  |
| *ARG1*  (Human) | F: ACTGACAACCACAAGTGGA | qRT-PCR |
|  | R: GCACATCGGGAATCTTTCC |  |
| *IL10*  (Human) | F: AAGACCCAGACATCAAGGC | qRT-PCR |
|  | R: AAGAAATCGATGACAGCGC |  |
| *IL1R2*  (Human) | F: TTTCTGCCTTCACCCTTCAG | qRT-PCR |
|  | R: GGCACCTCAGGGCTACAG |  |
| *18sRNA*  (Human) | F: GGAGTATGGTTGCAAAGCTGA | qRT-PCR |
|  | R: ATCTGTCAATCCTGTCCGTGT |  |
| *CCL2*  (Human) | F: GCCCCAGTCACCTGCTGTTAT | qRT-PCR |
|  | R: CTGCTTGGGGTCAGCACAGA |  |
| *CSF1*  (Human) | F: AACAGTTGAAAGATCCAGTGTG | qRT-PCR |
|  | R: TATCTCTGAAGCGCATGGT |  |
| *GAPDH*  (Human) | F: CCACATCGCTCAGACACCAT | qRT-PCR |
|  | R: GCGCCCAATACGACCAAAT |  |
| *Albumin*  (Mouse) | F: TGACCCAGTGTTGTGCAGAG | qRT-PCR |
|  | R: TTCTCCTTCACACCATCAAGC |  |
| *Cytoglobin*  (Mouse) | F: CCTGGTGAGGTTCTTTGTG | qRT-PCR |
|  | R: CATCTCCAAGGGATCCTCC |  |
| *Gfap*  (Mouse) | F: CTCGTGTGGATTTGGAGAG | qRT-PCR |
|  | R: GTTCTCGAACTTCCTCCTC |  |
| *Ash1l*  (Mouse) | F: CACAAATTGTAGCCCTACTCG | qRT-PCR |
|  | R: TGATGTACTATCCGCTGCAAC |  |
| *Kmt2a*  (Mouse) | F: AGGGAAGCTCCAAATAGGAAGG | qRT-PCR |
|  | R: GGGTCTTTATCCGTTCTGTGG |  |
| *Kmt2c*  (Mouse) | F: ACACCTGGACTTACCTTACCTT | qRT-PCR |
|  | R: TCGTGGAGCGTAGTTCTGTATT |  |
| *Setd1a*  (Mouse) | F: TTCCAGTGGCGGAACTACAAG | qRT-PCR |
|  | R: AACCGGGAGGGAAAAGTCTCT |  |
| *Smyd3*  (Mouse) | F: CCGACCCCTTGGCTTACAC | qRT-PCR |
|  | R: CATTGAGAACAACGCATCAGC |  |
| *Cd163* | F: TCAGCGACTTACAGTTTCCT | qRT-PCR |
| (Mouse) | R: TCATCCGCCTTTGAATCCA |  |
| *Il10*  (Mouse) | F: TTAATAAGCTCCAAGACCAAGG | qRT-PCR |
|  | R: CATCATGTATGCTTCTATGCAG |  |
| *Cd206*  (Mouse) | F: TACACAAATTCAGGGTTCTGG | qRT-PCR |
|  | R: GATGCTGCTGTTATGTCTCTG |  |
| *Tgfb1*  (Mouse) | F: TGCGCTTGCAGAGATTAAAA | qRT-PCR |
|  | R: CTGCCGTACAACTCCAGTGA |  |
| *18sRNA*  (Mouse) | F: GCTGGAATTACCGCGGCT | qRT-PCR |
|  | R: CGGCTACCACATCCAAGGAA |  |
| *Gapdh*  (Mouse) | F: AGGTCGGTGTGAACGGATTTG | qRT-PCR |
|  | R: TGTAGACCATGTAGTTGAGGTCA |  |
| *Ccl2*  (Mouse) | F: GGGCCTGCTGTTCACAGTT | qRT-PCR |
|  | R: CCAGCCTACTCATTGGGAT |  |
| *Csf1*  (Mouse) | F: CAAGGAGGTGTCAGAACAC | qRT-PCR |
|  | R: CATTTGACTGTCGATCAACTG |  |
| *CCL2*  (Human) | F: GCTCAGCAGATTTAACAGCC | ChIP-qPCR |
|  | R: GCTGCTGTCTCTGCCTCTTA |  |
| *CSF1*  (Human) | F: GTGCCTTGAAGTGTCTGC | ChIP-qPCR |
|  | R: CCATTCTCGGAGTGCC |  |
| sh-ASH1L#1  (Human) | CGTCTACGAAAGGCCTATTAC | shRNAs |
| sh-ASH1L#2  (Human) | GGATGATTGAGCAGTATCATA | shRNAs |
| si-ASH1L#1  (Mouse) | CGCTTACGAAAGGCCTATTAC | siRNAs |
| si-ASH1L#2  (Mouse) | GGATGATTGAGCAATATCATA | siRNAs |

**Supplementary Table S2. List of antibodies for flow cytometry analysis**

| **Antibody** | **Manufacturer** | **Catalog number** |
| --- | --- | --- |
| FITC Rat Anti-Mouse CD45 | BD | 553079 |
| Alexa Fluor® 488 anti-mouse CD3 Antibody | Biolegend | 135517 |
| PerCP-Cy™5.5 Rat Anti-Mouse CD4 | BD Bioscience | 550954 |
| Brilliant Violet 605™ anti-mouse CD8a Antibody | BioLegend | 100744 |
| PE/Cyanine7 anti-mouse NK-1.1 Antibody | BioLegend | 156514 |
| PE anti-mouse CD19 Anti-body | BioLegend | 115507 |
| BV510 Rat Anti-CD11b | BD | 562950 |
| Brilliant Violet 650™ anti-mouse CD11c | Biolegend | 117339 |
| Brilliant Violet 510™ anti-mouse I-A/I-E Antibody | Biolegend | 107635 |
| Alexa Fluor® 700 anti-mouse F4/80 | Biolegend | 123130 |
| APC anti-mouse CD3e Antibody | eBioscience | 17-0031-81 |
| PE/Cyanine7 anti-mouse CD8a Antibody | Biolegend | 100721 |
| CFSE Cell Division Tracker Kit | Biolegend | 423801 |
| **Reagent** |  |  |
| DAPI Solution | eBioscience | 564907 |
| Red Cell Lysis Buffer | TIANGEN | RT122-02 |
| Commercial Kits |  |  |
| Zombie NIR™ Fixable Viability Kit | Biolegend | 423106 |
| Tumor Dissociation Kit, mouse | MILTENYI | 130-096-730 |
| **Software and algorithms** |  |  |
| Software and algorithms | FlowJo | www.flowjo.com |
